# Induction of macrophage efferocytosis in pancreatic cancer via PI3Kγ inhibition and radiotherapy promotes tumour control

**DOI:** 10.1136/gutjnl-2024-333492

**Published:** 2025-01-09

**Authors:** Shannon Nicole Russell, Constantinos Demetriou, Giampiero Valenzano, Alice Evans, Simei Go, Tess Stanly, Ahmet Hazini, Frances Willenbrock, Alex Nicolas Gordon-Weeks, Somnath Mukherjee, Matthias Tesson, Jennifer P Morton, Eric O'Neill, Keaton Ian Jones

**Affiliations:** 1 Department of Oncology, University of Oxford, Oxford, UK; 2 Nuffield Department of Surgical Sciences, University of Oxford, Oxford, UK; 3 Institute of Cancer Sciences, CRUK Scotland Institute, Glasgow, UK

**Keywords:** CANCER, MACROPHAGES, PANCREATIC CANCER, ANTIGEN PRESENTATION

## Abstract

**Background:**

The immune suppression mechanisms in pancreatic ductal adenocarcinoma (PDAC) remain unknown, but preclinical studies have implicated macrophage-mediated immune tolerance. Hence, pathways that regulate macrophage phenotype are of strategic interest, with reprogramming strategies focusing on inhibitors of phosphoinositide 3-kinase-gamma (PI3Kγ) due to restricted immune cell expression. Inhibition of PI3Kγ alone is ineffective in PDAC, despite increased infiltration of CD8+ T cells.

**Objective:**

We hypothesised that the immune stimulatory effects of radiation, and its ability to boost tumour antigen availability could synergise with PI3Kγ inhibition to augment antitumour immunity.

**Design:**

We used orthoptic and genetically engineered mouse models of pancreatic cancer (LSL-Kras^G12D/+^;Trp53^R172H/+^;Pdx1-Cre). Stereotactic radiotherapy was delivered using contrast CT imaging, and PI3Kγ inhibitors by oral administration. Changes in the tumour microenvironment were quantified by flow cytometry, multiplex immunohistochemistry and RNA sequencing. Tumour-educated macrophages were used to investigate efferocytosis, antigen presentation and CD8+ T cell activation. Single-cell RNA sequencing data and fresh tumour samples with autologous macrophages to validate our findings.

**Results:**

Tumour-associated macrophages that employ efferocytosis to eradicate apoptotic cells can be redirected to present tumour antigens, stimulate CD8+ T cell responses and increase local tumour control. Specifically, we demonstrate how PI3Kγ signalling restricts inflammatory macrophages and that inhibition supports MERTK-dependent efferocytosis. We further find that the combination of PI3Kγ inhibition with targeted radiotherapy stimulates inflammatory macrophages to invoke a pathogen-induced like efferocytosis that switches from immune tolerant to antigen presenting.

**Conclusions:**

Our data supports a new immunotherapeutic approach and a translational rationale to improve survival in PDAC.

**WHAT IS ALREADY KNOWN ON THIS TOPIC**

- Radiotherapy can stimulate antitumour immunity, but meaningful responses are unpredictable and are frequently limited by simultaneous immune suppression.
- Macrophages can be repolarised via inhibition of the phosphoinositide 3-kinase-gamma (PI3Kγ) pathway, but this has little impact on the growth of pancreatic cancer.
- Macrophage efferocytosis induces a wound-repair like phenotype that suppresses immunity and promotes tumour growth.

**WHAT THIS STUDY ADDS**

- The immune stimulatory effects of radiotherapy can be exploited by concurrent PI3Kγ inhibition to significantly constrain pancreatic cancer growth.
- Combined radiotherapy and PI3Kγ inhibition stimulate macrophage efferocytosis of tumour cells by macrophages but not dendritic cells.
- Inhibition of PI3Kγ facilitates macrophage antigen presentation and prevents M2 polarisation resulting in a robust CD8+ T cell mediated antitumour response.

**HOW THIS STUDY MIGHT AFFECT RESEARCH, PRACTICE OR POLICY**

- These observations provide justification for clinical trials to test the effect of PI3Kγ inhibition combined with hypofractionated radiotherapy for pancreatic cancer.

## Introduction

 Immunotherapies are providing impressive survival benefits for patients across multiple tumour types, yet pancreatic cancer remains completely refractory to immune intervention. Macrophages play a leading role in both tumour control through inflammation, and resistance by promoting immune tolerance which in cancer creates an immune suppressed environment that supports tumour growth. As a result, the dual goal of eliminating tumour promoting macrophages while bolstering antitumour macrophages has become highly desirable for the potential to revert suppressive immune microenvironments and increase tumour control. Hence, pathways that regulate cell phenotype such as phosphoinositide 3-kinase (PI3K) are of particular strategic interest, with macrophage reprogramming strategies focusing on inhibitors of PI3K-gamma (PI3Kγ) due to restricted immune cell expression.

PI3Ks are essential for key cellular processes, including proliferation, survival and motility, and are expressed across nearly all cell types. The class IB isoform, PI3Kγ, plays a pivotal role in shifting tumour-associated macrophages (TAMs) to an immunosuppressive phenotype.^1^ Genetic or pharmacological inhibition of PI3Kγ can repolarise TAMs, reducing tumour immune suppression and permitting CD8+ T cell infiltration.^2^ However, targeting TAMs with PI3Kγ inhibition alone is insufficient to control tumour growth, with the greatest benefits typically seen when it is combined with other therapies.^3^ In genetically engineered models of pancreatic cancer, PI3Kγ has been shown to be essential for tumour progression, but despite this pharmacological treatment of established tumours has shown limited effectiveness.^4^

Patients harbouring cancers with a higher tumour mutational burden (TMB) are known to be more sensitive to immunotherapy.^5 6^ Consistent with this observation, the small subset (<3%) of pancreatic cancer patients with a higher TMB survive significantly longer following treatment with immune checkpoint inhibitors.^7^ Radiotherapy has previously been shown to improve the availability of tumour antigens, rationalising its use as an adjunct to improve sensitivity to immunotherapy.^8^ However, despite striking benefits in some solid tumours, radio-immunotherapy combinations have failed to translate in the treatment of pancreatic cancer.^9^ Evidence from preclinical models suggests that the immune stimulatory benefits of radiotherapy may be overcome by the recruitment and polarisation of myeloid cells, an effect is largely driven by the upregulation of cytokines and chemokines.^10 11^ However, radiation can also stimulate innate immunity via the release of damage-associated molecular patterns (DAMPs). Therefore, by preventing polarisation PI3Kγ inhibition could potentially synergise with radiotherapy to exacerbate the inflammatory capacity of macrophages in response to these signals.

We tested the effect of radiotherapy combined with PI3Kγ inhibition on tumour immunity and survival in high fidelity models of pancreatic cancer. We demonstrate that combined treatment constrains primary tumour progression and extends survival in a CD8+ T cell dependent manner. Strategies aiming to increase phagocytotic antitumour macrophages receive considerable attention for the potential to increase antigen presentation, induce inflammatory cascades and drive immunological control of tumours. Unexpectedly, we find that PI3Kγ inhibition does not involve phagocytosis but increases the clearance of radiation induced apoptotic tumour cells by macrophages via efferocytosis, a physiological cell clearance process that is usually immunologically silent. Efferocytosis is classically associated with polarisation to an immunosuppressive phenotype in macrophages and tolerance of self-antigens, a necessary physiological response to prevent autoimmunity. However, pathogen infected apoptotic cells are efferocytosed by dendritic cells leading to antigen presentation via both MHCII and MHCI, supported by pathogen associated molecular patterns and TLR signalling. Radiotherapy of tumours not only increases the antigen/neoantigen repertoire but also elevates DAMP-mediated TLR signalling.^12^ Notably, we find the unique combination of irradiation and PI3Kγ inhibition retains an inflammatory phenotype in efferocytic macrophages, rather than DCs, and also improves their capacity to present tumour antigens and prime CD8+ T cells. Ultimately, our data supports the exploitation of an endogenous cell-clearance mechanism to promote beneficial tumour control by employing targeted radiotherapy and myeloid specific PI3K inhibition.

## Methods

Detailed methods are described in online supplemental methods.

## Results

### PI3Kγ is expressed in human pancreatic cancer associated macrophages

**Figure 1:**
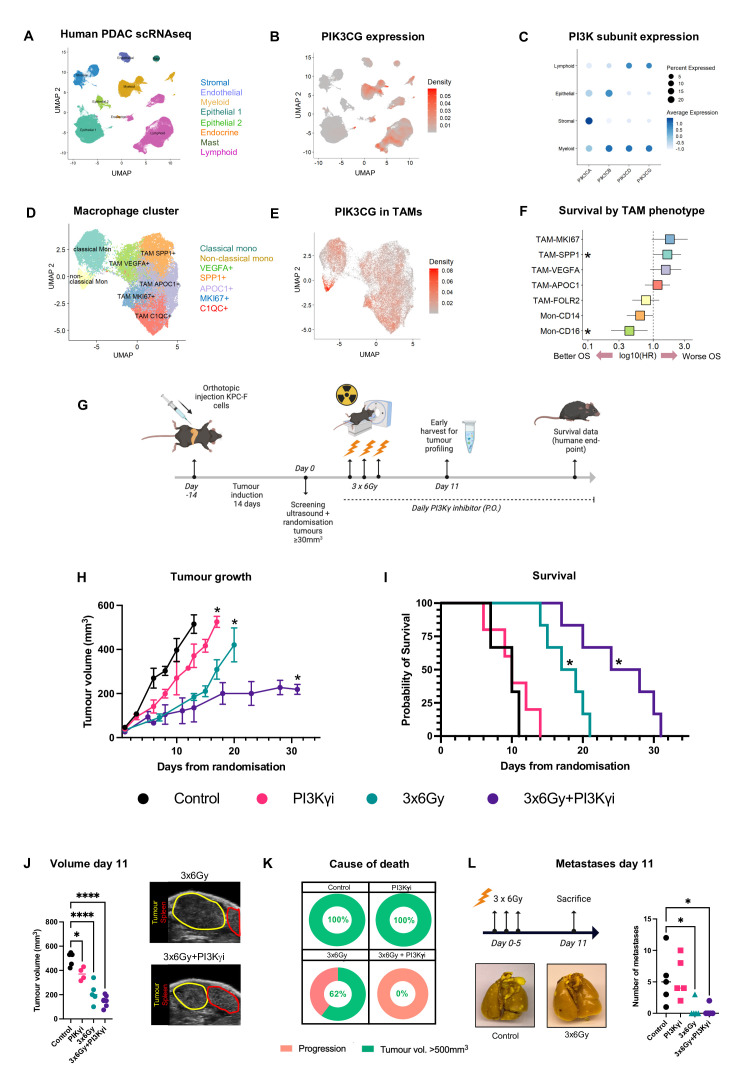
IR combined with PI3Kγ inhibition constrains primary tumour growth but does not affect metastasis. (**A,B**) Single cell transcriptomic data from PDAC patients was used to determine UMAP plots of their (**A**) cellular identity and (**B**) corresponding PIK3CG expression. (**C**) Expression of selected PI3K isoforms across different cellular compartments. (**D,E**) UMAPs derived from scRNAseq analysis illustrating (**D**) macrophage phenotype and (**E**) their corresponding PI3KCG expression. (**F**) Forest plot displaying the results of a multivariate Cox regression analysis testing the effect macrophage clusters identified in patients on disease-free survival. Covariates include age, sex, tumour stage and prior radiation therapy. (**G**) Schematic outlining the experimental design for testing the effect of different treatments in orthotopically implanted tumours. (**H**) Kaplan-Meier survival plot of mice bearing orthotopic KPC tumours as treated in G; log-rank Mantel-Cox test, p<0.05). Representative example of n=3 independent experiments. (**I**) Tumour growth kinetics of mice bearing orthotopic KPC tumours as treated in (G). Analysed by one‐way analysis of variance (ANOVA) with Tukey’s post hoc adjustment (n=5–6 mice/group). Representative example of n=3 independent experiments. (**J**) Comparison of tumour volume 11 days following randomisation measured by 3D ultrasound. Representative ultrasound images are shown. Analysed by one‐way ANOVA with Tukey’s post hoc adjustment (n=4–7 mice/ treatment). (**K**) Cause of death of mice across the four treatment groups in mice with orthotopic tumours defined as either humane end-point (tumour >500 mm^3^) or disease progression. (**L**) Lungs were harvested 11 days following randomisation and metastases were quantified for each treatment group (n*=*5). Representative macroscopic images of isolated lungs stained with Bouin’s solution. Analysed by one‐way ANOVA with Tukey’s post hoc adjustment (n=4–7 mice/ treatment). *p<0.05, **p<0.01, ***p<0.001. Data presented as mean±SEM. PDAC, pancreatic ductal adenocarcinoma; PI3Kγ, phosphoinositide 3-kinase-gamma; TAM, tumour-associated macrophage.

Using scRNA-seq data from human pancreatic cancer patients, we examined the differential expression of the major PI3K isoforms across different cell populations (figure 1A–C, online supplemental figure 1A,B). Expectedly, PI3Kα and PI3Kβ are abundant in epithelial cells and fibroblasts, neither of which express PI3Kγ (online supplemental figure A,B). Notably, PI3Kγ expression is not restricted to myeloid cells, but co-expressed with PI3Kδ in other immune cells including T cells, natural killer cells, B cells and Tuft cells (online supplemental figure 1A). The macrophage lineage was further divided into predominant populations: suppressive (SPP1+, APOC1+), pro-tumour (VEGFA+, MKI67+), inflammatory (C1QC+), classical (CD14+) and non-classical cells (CD16+) (figure 1D). PI3Kγ is ubiquitously expressed across all populations, notably with an enrichment in the non-classical monocyte group (CD16+; figure 1E). Next, we explored cell-cell communication between cells in the pancreatic ductal adenocarcinoma (PDAC) microenvironment which reveals a strong correlation between tumour macrophages and CD8+ T cells, emphasising the cross-talk between potentially suppressive TAMs and effector T cells (online supplemental figure 1C,D). We further explored the role of TAMs by measuring their impact on survival outcomes. Despite controlling for age, gender, stage and treatment status, we find that specific macrophage signatures significantly impact survival. Macrophage signatures associated with worse survival include proliferating (MKi67+), osteopontin-high (SPP1+) and angiogenic (VEGFA+) markers, while classical and non-classical monocyte signatures correlate with improved survival (figure 1F).

### Combined treatment constrains primary tumour growth and extends survival

To address whether reprogramming of the myeloid compartment by PI3Kγ contributes to tumour control, we employed the previously described KPC-F cell line derived from an *LSL-Kras^G12D/+^;Trp53^R172H/+^;Pdx1-Cre* C57BL/6 tumour to generate orthoptic tumours^13^ (figure 1G). We find PI3Kγ inhibition alone does not significantly affect tumour growth or survival, suggesting that modulating the immune microenvironment in isolation is insufficient (figure 1H,I). However, whereas radiotherapy (IR) modestly improves survival, combination with PI3Kγ inhibition constrains tumour growth and extends survival (figure 1H–J).

We noticed that IR treated animals reached endpoint with a high proportion of primary tumours displaying better local control and remaining below 500mm^3^ (figure 1K). Compared with animals treated with IR alone, IR+PI3Kγ inhibition increases the extent of local control (38% IR, 100% IR+PI3Kγ inhibitor), suggesting that mice were not reaching endpoint because of primary tumour progression but potentially from disseminated disease. When quantified, mice treated with IR+PI3Kγ inhibitor succumbed more frequently due to progressive disease (figure 1K). This implies that the combination either stimulates metastasis or that the extended period of survival affords the opportunity for metastases to progress. To address this directly, we quantified metastatic burden at a fixed early time point after treatment. We find evidence of widespread dissemination in untreated animals, however, in mice receiving IR, there were fewer metastases overall suggesting improved local control delays the onset of symptomatic metastases (figure 1L, online supplemental figure 2A,B).

**Figure 2:**
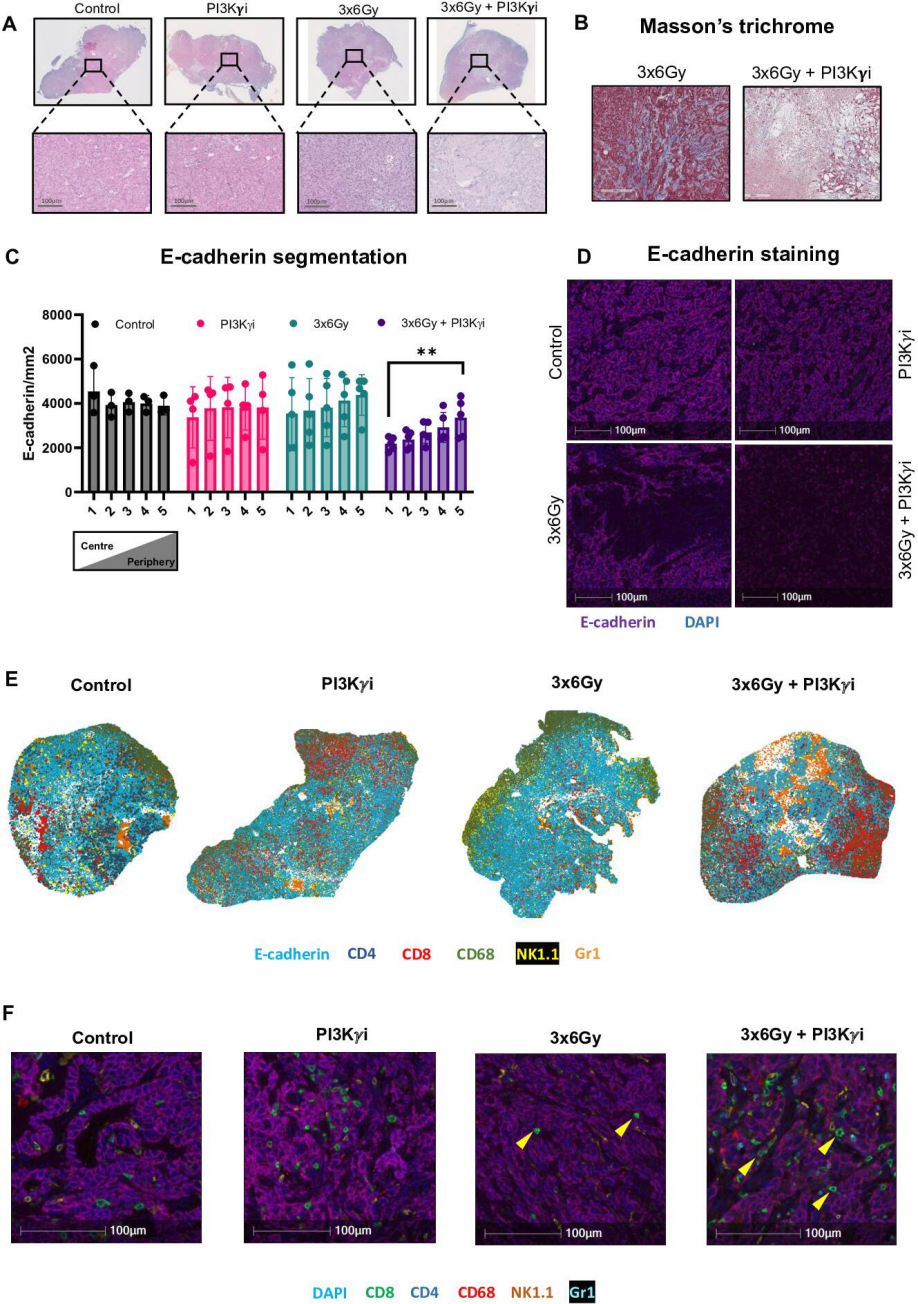
IR combined with PI3Kγ inhibition reduces tumour epithelial cellularity and improves CD8+ T cell infiltration (**A**) Representative H&E-stained f orthotopic KPC-F tumour sections across each treatment group. (**B**) Mason’s trichrome staining of orthotopic KPC-F tumour sections from 3×6 Gy and 3×6Gy+PI3Kγ inhibitor treated animals. (**C**) E-cadherin was quantified in serial segments of tumour slices from the centre to the periphery (1–5) across each treatment group. Analysed by one‐way analysis of variance (ANOVA) with Tukey’s post hoc adjustment (n*=*3–5 mice/ group). (**D**) Immunofluorescent staining of KPC-F tumour sections across each treatment group. Purple=E-cadherin, blue=DAPI. (**E**) Representative spatial plots showing individual cell populations segmented using HALO machine-learning software. Data generated from multiplex immunofluorescent staining of KPC-F tumour sections across each treatment group. Cyan=E-cadherin, red=CD8, blue=CD4, green=CD68, yellow=NK1.1, orange=Gr-1. (**F**) Representative multiplex immunofluorescent images of treated tumour samples across treatment groups. Cyan=DAPI, purple=E-cadherin, green=CD8, blue=CD4, red=CD68, orange=NK1.1. Sections presented in panel A are identical to those in figure 3G but with all fluorescent channels and markers displayed. *p<0.05, **p<0.01, ***p<0.001. PI3Kγ, phosphoinositide 3-kinase-gamma.

We also tested IR±PI3Kγ inhibition in a GEM model of pancreatic cancer (*LSL-Kras^G12D/+^;Trp53^R172H/+^;Pdx1-Cre* C57BL/6). We find that primary tumours are constrained by PI3Kγ inhibition, though this does not translate to a survival advantage (online supplemental figure 2C,D). Experimentally, GE mice were randomised with larger tumours, which we find has a direct impact on IR response and survival (online supplemental figure 2E). Overall, these data suggest that while combined treatment effectively constrains primary tumour progression and delays the onset of systemic disease, it does not reduce the ultimate incidence of metastasis.

### IR combined with PI3Kγ inhibition reduces tumour epithelial cellularity

Despite improved local control in the combined treatment group, tumour volume remained static. Therefore, we sought to examine the histological effect in tumours in response to treatments. Striking differences are apparent in the centre of the tumours with a substantial loss of epithelial cells and altered stromal architecture in the combination treatment group (figure 2A,B). Consistently, there is a significant loss of E-cadherin+ cells in the centre of combination treated tumours (figure 2C,D). We next quantified immune cells using multiplex immunohistochemistry (online supplemental figure 3A). Analysis of tumour infiltration by segmentation reveals a general trend towards fewer immune cells in central regions, though this is not statistically significant (online supplemental figure 3C,F). Comparable to other immune cells, we observed fewer CD8+ T cells in the tumour centre (figure 2E, online supplemental figure 3G). However, topographical assessment of whole tumour sections from PI3Kγ treated mice revealed that CD8+ T cells appear to be present in areas of viable tumour (figure 2E). Quantitatively, there are more CD8+ T cells present in E-cadherin rich areas of combination treated tumours (online supplemental figure 3G). High-magnification images reveal that in IR-treated tumours, CD8+ T cells localise within the stroma, whereas in combination-treated tumours, they appear in direct contact with E-cadherin+ cells (figure 2F). These observations indicate that although adaptive immunity is engaged in response to combined treatment with necrosis and reduced tumour cellularity, tumour volume is unaffected.

**Figure 3:**
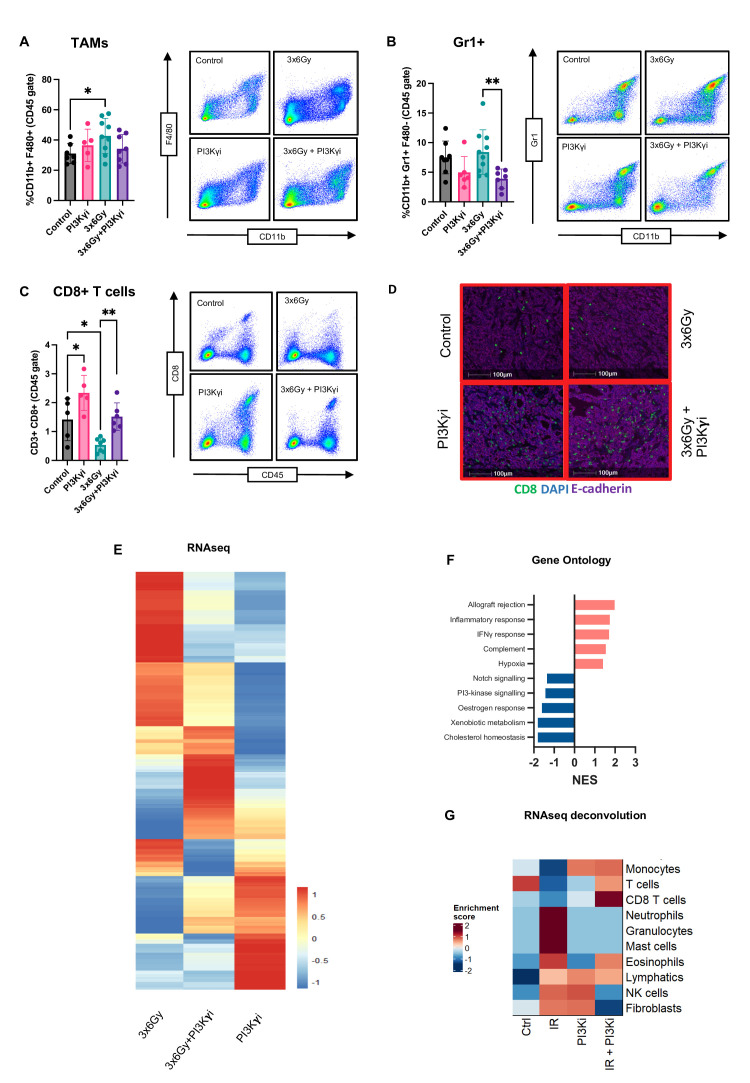
Combined treatment alters innate and adaptive immune responses within the tumour-educated macrophage. (**A**) Flow cytometric analysis of tumour-associated macrophages (CD11b+F4/80+) KPC-F tumours receiving indicated treatments. Analysed by one‐way analysis of variance (ANOVA) with Tukey’s post hoc adjustment (n=5–9/group). Representative example of flow cytometry analysis of tumours from n=3 independent animal experiments. (**B**) Flow cytometric analysis of MDSCs (CD11b+F4/80- Gr-1+) in KPC-F tumours receiving indicated treatments. Analysed by one‐way ANOVA with Tukey’s post hoc adjustment (n=5–9/group). Representative example of flow cytometry analysis of tumours from n=3 independent animal experiments. (**C**) Flow cytometric analysis of CD8+ T cells (CD3+CD8+) in KPC-F tumours receiving indicated treatments. Analysed by one‐way ANOVA with Tukey’s post hoc adjustment (n=5–9/group). Representative example of flow cytometry analysis of tumours from n=3 independent animal experiments. (**D**) Representative immunofluorescent staining images of tumours sections from mice receiving treatments as indicated (purple=e-cadherin; blue=DAPI, green=CD8). (**E**) Bulk RNAseq analysis of tumours from each treatment group. A histogram of the log2fold change of these genes between each treatment group and the control group is shown (n*=*3). (**F**) Gene ontology analysis of top five upregulated and downregulated differentially expressed pathways between the IR versus IR+PI3Kγi. Pathways significant at p<0.05 are presented. (**G**) Immune deconvolution of each treatment group was performed on bulk tumour RNAseq data and subsequently plotted as a histogram. *p<0.05, **p<0.01, ***p<0.001. Data presented as mean±SEM. PI3Kγ, phosphoinositide 3-kinase-gamma.

### Combined treatment alters innate and adaptive immune responses within the TME

To obtain a detailed quantification of total and relative immune cell populations, we analysed dissociated tumours by flow cytometry. In response to IR, tumours exhibit a significant increase in macrophage numbers, though their phenotype remains unchanged, as indicated by their stable iNOS (M1) ratio (figure 3A; online supplemental figure 3H). PI3Kγ inhibition independently increases the iNOS+ ratio, regardless of concurrent IR treatment. While total macrophage counts are unaffected by PI3Kγ inhibition, there is a notable reduction in CD11b+Gr1+ cells and type 1 conventional dendritic cells (figure 3B, online supplemental figure 3I). Further analysis of Gr1+ cells by Ly6C/Ly6G expression shows that the monocytic subset (Ly6C^hi^/Ly6G^lo^) comprises the majority of the total population and increases following IR. With PI3Kγ inhibition, Gr1+ cells decrease to levels comparable to those in untreated tumours (online supplemental figure 3J). However, prior immunohistochemical analysis of Gr1+ cell distribution revealed a higher density in tumours receiving combination therapy (figure 2E). These cells spatially cluster in necrotic regions, suggesting they may represent Ly6G+ granulocytic cells identifiable by anti-Gr1 antibodies. Subsequent quantification of Ly6G(1A8)+ cells by immunohistochemistry confirmed a substantial increase in necrotic regions of combination-treated tumours, corresponding with Gr1 staining patterns in the multiplex panel (online supplemental file 4). These findings suggest that while monocytic Ly6Chi Gr1+ cells decrease in response to combination therapy, Ly6Ghi Gr1+cells redistribute and infiltrate necrotic areas.

**Figure 4:**
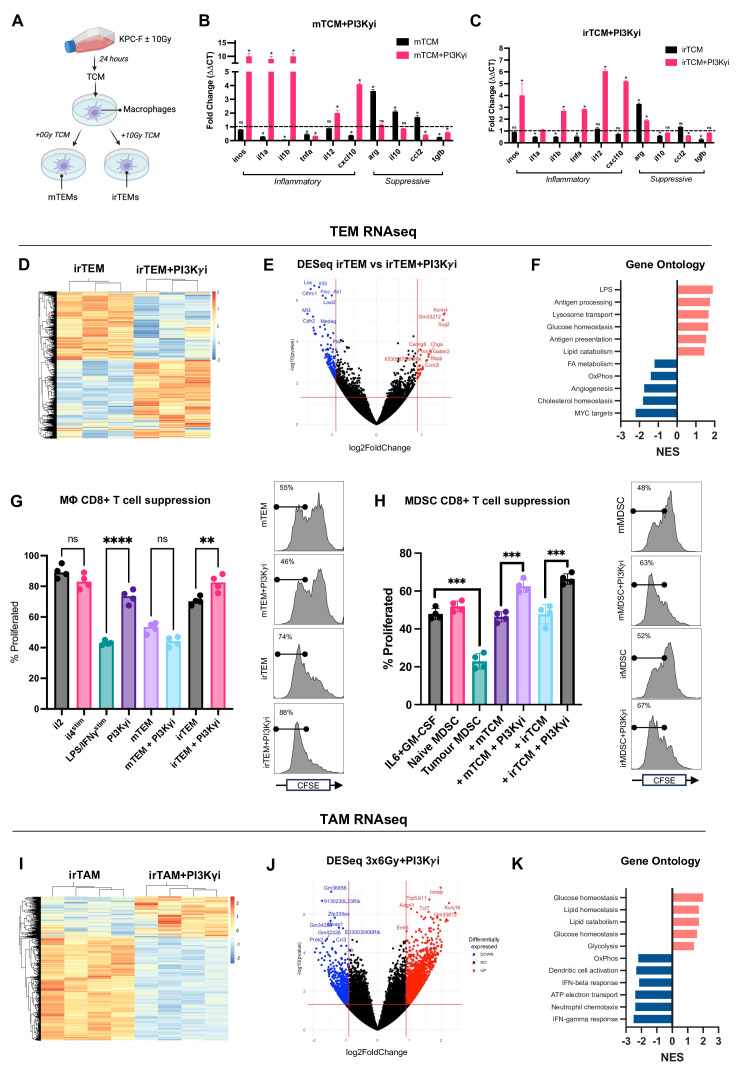
Treatment with IR+PI3Kγ inhibition alters macrophage metabolism. (**A**) Schematic illustrating the workflow of producing tumour-educated macrophages (TEMs). (B+C) TEMs were generated as outlined in (**A**) in the (**B**) absence or (**C**) presence of PI3Kγ inhibitor, with expression of selected immune stimulatory and immunosuppressive genes analysed via RT-qPCR. Analysed by Mann-Whitney test. (**D**) Histogram of the log2fold change of these genes in irTEM±PI3Kγ inhibitor is shown (n*=*3 per condition). (**E**) Volcano plot comparing transcriptomic changes in irTEM±PI3Kγ inhibitor. Genes represented by blue (downregulated) and red dots (upregulated) are those with padj.<0.05. (**F**) Gene ontology analysis of top five upregulated and downregulated differentially expressed genes between irTEM±PI3Kγ inhibitor. Pathways significant at p<0.05 are presented. (G+H) The ability of (**G**) TEMs and (**H**) MDSCs to suppress CD8+ proliferation was quantified via flow cytometry. Primary murine macrophages and MDSCs were differentiated in vitro and exposed to LPS+IFNγ (**M1**), interleukin 4 (**M2**), tumour conditioned media (as indicated)±PI3Kγ inhibitor. Macrophages were then co-cultured with CFSE stained CD8+ T cells and proliferation assessed after 72 hours by flow cytometry. Data are presented as individual values with the horizontal bar representing the mean value. Statistical significance between PI3Kγ inhibitor and vehicle treated samples by two-tailed, one sample t-tests (n*=*3–4 per condition). Representative data from n=2 independent experiments. (**I**) RNAseq analysis of CD11b+ cells, with statistically significant differentially expressed genes identified and isolated. A histogram of the log2fold change of these genes in irTAM±PI3Kγ inhibitor is shown (n=4 per condition). (**J**) Volcano plot comparing transcriptomic changes in irTAM±PI3Kγ inhibitor. Genes represented by blue (downregulated) and red dots (upregulated) are those with padj.<0.05. (**K**) Gene ontology analysis of top five upregulated and downregulated differentially expressed genes between irTAM±PI3Kγ inhibitor. Pathways significant at p<0.05 are presented. *p<0.05, **p<0.01, ***p<0.001. PI3Kγ, phosphoinositide 3-kinase-gamma.

Analysis of lymphocytes reveals a significant IR-induced reduction in CD8+ T cells, which is restored with the addition of PI3Kγ inhibition (figure 3C,D). Notably, the relative increase in CD8+ T cells is ~60% in both groups receiving PI3Kγ inhibition. Although the absolute number of CD8+ T cells in combination treated tumours is lower than the PI3Kγ monotherapy group, they are more cytotoxic with elevated expression of granzyme B, perforin and TIGIT, indicating a checkpoint-engaged phenotype (online supplemental figure 3K). These results demonstrate that combined treatment elicits a more potent reduction in suppressive immune cells coupled to an increase in the number of activated effector T cells when compared with PI3Kγ inhibition or IR alone. This suggests the two treatments are at least additive, with PI3Kγ inhibition potentiating the immune stimulatory effects of IR, while limiting suppressive activity.

### Gene ontology reveals upregulation of pathways associated with adaptive immunity

To validate observations made by flow cytometry and immunohistochemistry, which suggested that adaptive immunity was engaged, we analysed whole tumour samples by RNA sequencing. We find significant alterations in genes across all four treatment groups, with 6239 significantly differentially expressed genes in combination treated tumours compared with IR alone (figure 3E). Gene ontology analysis demonstrates significant upregulation of pathways associated with adaptive immunity and inflammation, including allograft rejection, inflammatory response and interferon-γ signalling (figure 3F). Digital cell quantification highlights that CD8+ T cells are the most significantly enriched population in combination treated tumours (figure 3G). These results support the conclusion that the effects of combined treatment on tumour growth are driven primarily through adaptive immunity activation.

### IR causes alterations in macrophage phenotype that are prevented by PI3Kγ inhibition

We tested the effect of PI3Kγ inhibition in the context of naïve macrophages exposed to tumour-conditioned media (TCM) from KPC cells treated with and without IR (‘mTEMs’ and ‘irTEMS’; figure 4A). We identified several significantly upregulated cytokines (M-CSF, IL-15 and PTX-3) present in the medium from irradiated KPC-F cells compared with non-irradiated cells, several of which are known to impact macrophage activation (online supplemental figure 5A).

**Figure 5:**
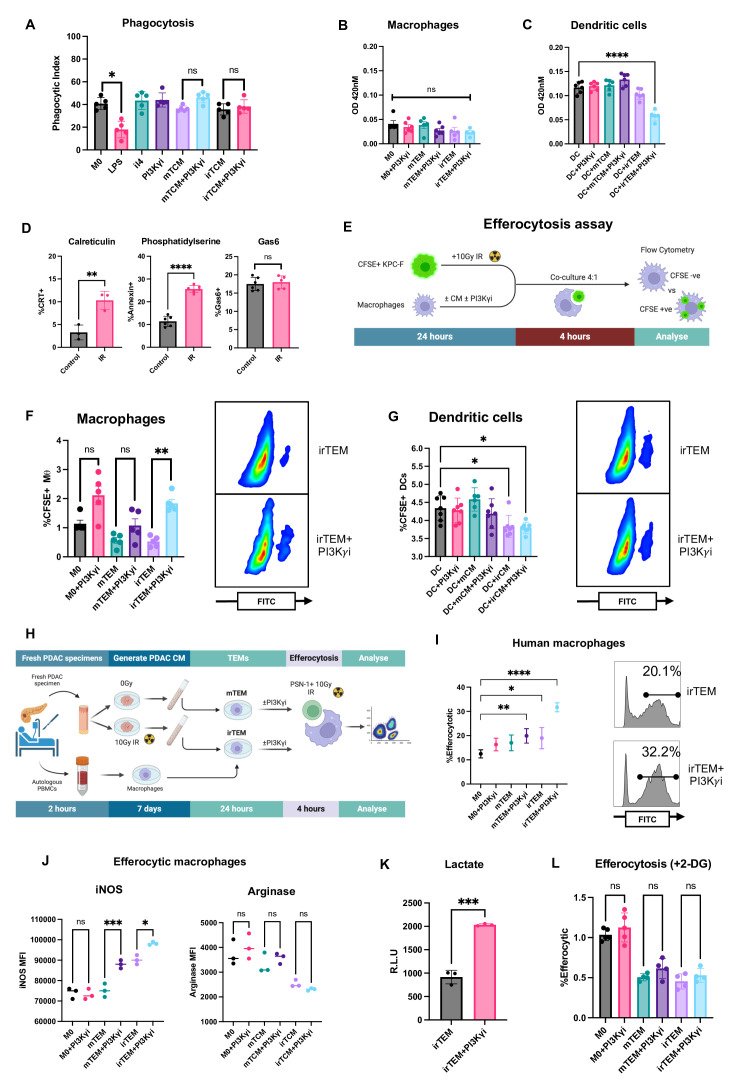
Treatment by irradiation and PI3Kγ inhibition increases the ability of macrophages to perform efferocytosis in both mice and humans. (**A**) Bioparticle phagocytosis in macrophages treated as indicated. Bone marrow-derived macrophages were exposed to LPS (**M1**), interleukin 4 (**M2**), tumour conditioned media±PI3Kγ inhibitor. Analysed by one‐way analysis of variance (ANOVA) with Tukey’s post hoc adjustment (n=5). Representative data from n=3 independent experiments. (**B,C**) Macrophages (**B**) and dendritic cells (**C**) were exposed to treatment conditions as indicated for 24 hours. Conditions were removed and OVALBUMIN coated beads were added and cells cultured for a further 4 hours. Beads were removed and cells co-cultured with B3Z T cells for 24 hours. B3Z T cell activation was quantified by measuring β-galactosidase activity colimetrically (optical density). Analysed by one‐way ANOVA with Tukey’s post hoc adjustment (n=4–5). Data representative of n=2 independent experiments. (**D**) Flow cytometric analysis of surface expression of calreticulin, phosphatidylserine and gas6 on untreated and IR treated KPC-F cells after 24 hours. Analysed by one‐way ANOVA with Tukey’s *post hoc* adjustment (n=4–5). (**E**) Schematic illustrating workflow of efferocytosis assay. KPC-F cells were irradiated (10 Gy) and stained with CFSE. Macrophages were exposed to indicated treatments including tumour cell conditioned media±PI3Kγ inhibitor. KPC-F tumour cells and macrophages were co-cultured for 4 hours at a ratio of 4:1 and subsequently analysed by flow cytometry. FITC+ macrophages were determined to be efferocytic. (**F,G**) Flow cytometric analysis of tumour-educated macrophages (TEMs) (**F**) and dendritic cells (**G**) co-cultured with irradiated KPC-F as per figure 5D. Representative flow cytometry plots are shown. Efferocytic cells were quantified by measuring total CFSE+ cells compared with CFSE− cells. Analysed by two-tailed, one sample t-tests (n=4–5). Representative of n=3 independent experiments. (**H**) Schematic illustrating the workflow for performing efferocytosis assay using samples obtained from human PDAC patients. Fresh tumour samples were embedded in agarose and precision cut using a vibratome. Slices were cultured treated with and without irradiation and conditioned media collected after 24 hours. Peripheral blood was collected from matched patients and CD14+ monocytes isolated by magnetic separation. Monocytes were cultured for 5 days in the presence of human M-CSF. Macrophages were exposed to tumour slice conditioned medium±PI3Kγ inhibitor for 24 hours. Pancreatic cancer PSN-1 cells were irradiated and stained with CFSE. Patient macrophages were co-cultured with matched macrophages for 4 hours at a ratio of 4:1 and subsequently analysed by flow cytometry. (**I**) Flow cytometric analysis of human TEMs co-cultured with irradiated CFSE labelled PSN-1 cells as per figure 5H. Analysed by one‐way ANOVA with Tukey’s post hoc adjustment (n=3). Data are representative of n=3 patient samples. (**J**) Quantification by flow cytometry of iNOS and Arginase expression in efferoyctic macrophages. Analysed by one‐way ANOVA with Tukey’s post hoc adjustment (n=3). (**K**) Culture media from macrophages receiving indicated treatments was collected and lactate concentration quantified by colorimetric assay. Data is presented as mean±SEM and analysed by two-tailed, one sample t-test (n*=*3). (**L**) Quantification of efferocytosis in macrophages receiving treatment as indicated. All groups received 2-DG to inhibit glycolysis. Efferocytic macrophages were quantified by measuring total CFSE+ macrophages compared with CFSE− macrophages (as per figure 5D). Data is presented as mean±SEM and analysed by one‐way ANOVA with Tukey’s post hoc adjustment (n*=*3). *p<0.05, **p<0.01, ***p<0.001. Data presented as mean±SEM. PI3Kγ, phosphoinositide 3-kinase-gamma.

To confirm whether these tumour secreted cytokines affected macrophage activation, we quantified changes in mRNA expression of several immune related genes. Inflammatory markers, such as Il1a, Il1b, Tnfa and Cxcl10, are downregulated in both mTEMs and irTEMs, while immunosuppressive markers including Arg, Il10 and Ccl2 are upregulated (figure 4B,C). Treatment with PI3Kγ inhibition reverses this phenotype, with reduced expression of immunosuppressive markers and increased expression of markers associated with inflammation (figure 4B,C). To confirm that these alterations translate into a functional phenotype, we assessed their capacity to suppress CD8+ T cell mediated tumour cell killing. Compared with naïve macrophages, mTEMs and irTEMs significantly suppress T cell killing, confirming their capacity to exert immune suppression (online supplemental figure 4B). Further interrogation by RNA sequencing reveals significant changes in irTEMs, with 2371 differentially expressed genes compared with naïve macrophages (online supplemental figure 4C). The most significantly upregulated genes, including Ccl2, Ccl3, Ccl6 and Ccl7, encode cytokines linked to a suppressive myeloid phenotype (online supplemental figure 4D). Ontological analysis shows upregulation in pathways associated with proliferation (E2F targets, G2M and MYC targets) and inflammation (NFκB, allograft rejection, interferon-γ response) (online supplemental figure 4E). The same analysis was used to detect changes in mRNA expression in irTEMs treated with PI3Kγ inhibition, where we find pathways involved in innate immunity are upregulated (lipopolysaccharide signalling, antigen processing; figure 4F). This observation supports the rationale for adding PI3Kγ inhibition to IR, leveraging immune-stimulatory signals induced by irradiation to enhance the antitumour activity of macrophages.

### CD8+ T cell suppression by TAMs and MDSCs is reversed by PI3Kγ inhibition

To account for the increased number of CD8+ T cells present in combination treated tumours, we tested the effect of TEMs on CD8+ T cell proliferation. As expected, untreated TEMs are capable of profoundly suppressing T cell proliferation (figure 4G). However, PI3Kγ inhibition alone does not reduce suppression in TEMs generated from untreated cell-conditioned media. Conversely, irTEMs are less suppressive and PI3Kγ inhibition potentiates this effect (figure 4G). The ubiquitous expression of PI3Kγ in myeloid cells indicated that MDSCs may likewise undergo reprogramming in response to PI3Kγ inhibition. We find that PI3Kγ inhibition reduces the suppressive capacity of MDSCs, and comparable to the results seen with TEMs there is a more pronounced effect in the irradiated group (figure 4H).

### IR and PI3Kγ inhibition maintains an inflammatory phenotype of tumour macrophages

To understand the effect of treatment on myeloid cell behaviour within the TME, we analysed RNA sequencing data from CD11b+ cells isolated from treated tumours. Gene ontology analysis reveals that IR upregulates pathways involved in antigen processing, presentation and acute myeloid inflammation (online supplemental figure 4I). There are more profound changes seen in response to IR+PI3Kγ inhibition, with 4067 differentially expressed genes (figure 4I,J). Notably, the most upregulated pathways involve glucose and lipid metabolism, while oxidative phosphorylation is significantly downregulated, suggesting a metabolic shift from oxidative phosphorylation towards glycolysis (figure 4K). This shift, commonly associated with an M1-like phenotype, often follows toll-like receptor engagement by DAMPs.^14^

Together, these data suggest that while IR promotes antigenicity, adaptive immunity may be thwarted by continued immune suppression within the TME. In contrast, myeloid cells in tumours treated with combined therapy exhibit an altered metabolic phenotype which is more pronounced in tumour myeloid cells compared with those treated in vitro. This indicates that unique pressures within the TME drive a novel macrophage phenotype specifically in response to combination therapy. The magnitude of change and differentially enriched pathways in TAMs compared with TEMs highlights the limitations of in vitro approaches to infer changes expected in vivo.

### PI3Kγ inhibition stimulates efferocytosis of irradiated tumour cells

In addition to the altered inflammatory and metabolic reprogramming indicated by the transcriptomic analyses, combined treatment with IR+PI3Kγ inhibition led to enrichment of pathways associated with antigen presentation in macrophages. Therefore, we next considered whether macrophages are more capable of presenting tumour antigens in their capacity as professional antigen presenting cells (APCs). Macrophages may endocytose foreign material via phagocytosis, typically associated with pathogenic infection, or efferocytosis, the clearance of apoptotic cells. To mimic phagocytosis, we quantified uptake of fluorescein-labelled *Escherichia coli (E. coli*) bioparticles, finding that TCM±PI3Kγ inhibition has no effect on the phagocytic capacity of macrophages (figure 5A). Next, we determined whether this correlated with antigen cross-presentation and T cell activation, directly comparing macrophages to dendritic cells. We exposed macrophages to iron oxide beads covalently bound to the model antigen OVALBUMIN (257-264) in the long-peptide form, which is restricted to MHC class I presentation. Importantly, beads were of comparable size to *E. coli* bioparticles (1.5 µM). As a readout of cross-presentation, we measured activation of the B3Z T cell hybridoma cell line as previously described.^15^ Consistent with the results of the phagocytosis assay, PI3Kγ inhibition does not affect direct antigen cross-presentation in macrophages, although we find impaired cross-presentation in dendritic cells exposed to irTCM+PI3Kγ inhibition (figure 5B,C). Expectedly, we confirmed that dendritic cells demonstrate higher levels of antigen cross-presentation compared with macrophages (OD 420 nM macrophages 0.075 vs dendritic cells 1.12). In the absence of an effect on phagocytosis in response to treatments, we next determined whether the ability of TEMs to engulf tumour cells via efferocytosis was altered.

Radiation can induce immunogenic cell death via DAMPs and TLR signalling, leading to the expression of ‘eat me’ signals including phosphatidylserine (PS), growth arrest-specific 6 (GAS6) and calreticulin (CRT).^16^ Engagement of the cognate receptors present on macrophages including TYRO3, AXL, MERTK ‘TAM’) can stimulate efferocytosis. We find that radiation significantly increases the surface expression of CRT and PS on tumour cells but has no effect on GAS6 (figure 5D). Strikingly, we find that irTEMs are significantly more efferocytic in response to PI3Kγ inhibition (figure 5E–F). Importantly, we do not see a similar response in dendritic cells exposed to identical conditions (figure 5G). Interestingly, using primary human pancreatic cancer samples and macrophages generated from autologous blood, the increase in efferocytosis previously seen in murine macrophage is replicated (figure 5H,I). Under normal physiological conditions, efferocytosis results in M2 polarisation. Therefore, we questioned whether efferocytic macrophages were polarised, and whether PI3Kγ inhibition prevented this. Both M1 and M2 markers increase following efferocytosis, indicative of generalised activation rather than polarisation (figure 5J). In response to PI3Kγ inhibition, efferocytic macrophages display higher levels of the M1 marker iNOS, and a trend towards lower levels of the M2 marker Arginase (p=ns), suggesting that the immunological silencing usually seen following efferocytosis is blocked by PI3Kγ inhibition (figure 5J). Finally, recent evidence suggests that efferocytosis induces transient aerobic glycolysis in macrophages.^17^,^19^ We found that glycolysis gene sets were enriched in TAMs but not TEMs, the major difference being exposure to tumour cells and the opportunity to efferocytose. Therefore, we questioned whether IR+PI3Kγ driven efferocytosis is associated with altered metabolism. By measuring lactate production, we find that efferocytic macrophages (irTEM+PI3Kγ inhibitor) are significantly more glycolytic compared with macrophages displaying less efferocytosis (irTEM) (figure 5K). Strikingly, the efferocytic capacity of TEMs is lost by inhibiting glycolysis, suggesting that the process creates an acute energy demand (2-DG; figure 5L)). Although the levels of efferocytosis are significantly increased following IR+PI3Kγ inhibition, the effect is modest (twofold increase). Therefore, we questioned whether the response is present in additional human and murine cell lines, and whether the magnitude of effect is comparable. We find that the baseline levels of efferocytosis is variable, ranging from ~3.5% in a murine melanoma cell line (B16-F10), to ~20% in a colon cancer line (MC38) (online supplemental figure 6A–F). Strikingly, a significant increase in efferocytosis in response to combined treatment is conserved across five of the six cell lines tested, with no difference observed in A549 cells. Together, these results indicate that efferocytosis is uniquely induced by IR and PI3Kγ inhibition and is not restricted to the KPC-F model. Critically, efferocytic macrophages retain an inflammatory phenotype in the context of PI3Kγ inhibition and require glycolysis to maintain this response.

**Figure 6:**
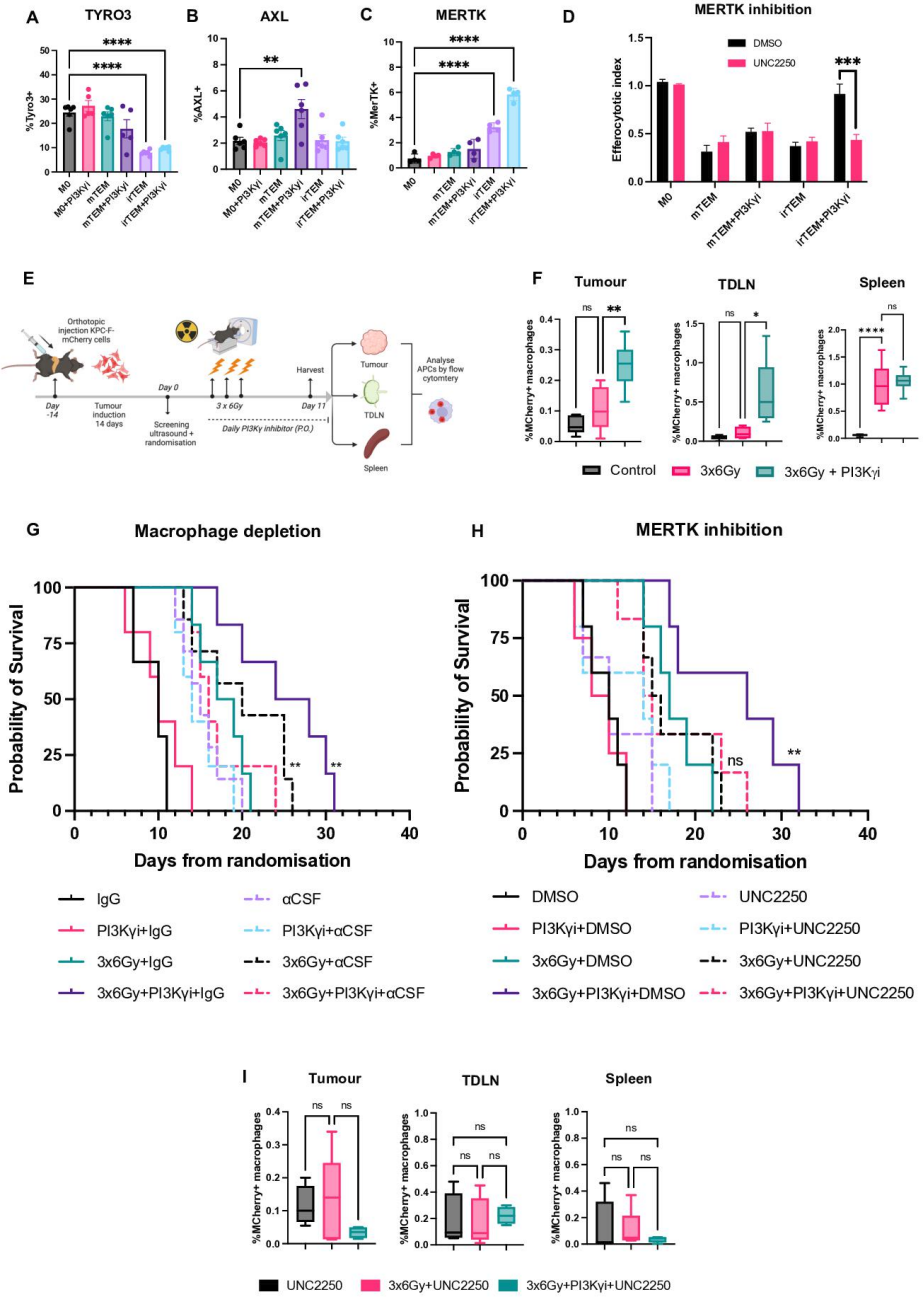
Treatment by irradiation and PI3Kγ inhibition leads to MERTK-dependent efferocytosis. (**A–C**) Flow cytometric analysis of TYRO3, AXL and MERTK expression on macrophages. Analysed by one‐way analysis of variance (ANOVA) with Tukey’s post hoc adjustment (n=5). (**D**) Quantification of efferocytosis in mouse macrophages exposed to conditioned as indicated. MERTK was inhibited by adding UNC2250 60 min prior to the co-culture. Analysed by one‐way ANOVA with Tukey’s post hoc adjustment (n=5). (**E**) Schematic illustration of the experimental protocol for quantifying efferocytic macrophages and dendritic cells in vivo. Briefly, KPC-F-mCherry cells were grown orthotopically and mice treated with IR±PI3Kγ inhibition. Five days following the final fraction of IR, tumours, draining lymph nodes and spleens were collected and mCherry+ cells quantified by flow cytometry. (**F**) Flow cytometric analysis of mCherry+ macrophages (CD45+CD11b+F4/80+) in tumour, tumour-draining lymph nodes (TDLN) and spleen. Analysed by one‐way ANOVA with Tukey’s post hoc adjustment (n=5 mice/ condition). (**G**) Kaplan-Meier survival plot of mice bearing orthotopic KPC tumours as treated in figure 1G±anti CSF; log-rank Mantel-Cox test, p<0.05. Experiment conducted once. (**H**) Kaplan-Meier survival plot of mice bearing orthotopic KPC tumours as treated in figure 1G±UNC2250; log-rank Mantel-Cox test, p<0.05. Experiment conducted once. (**I**) Flow cytometric analysis of mCherry+ macrophages (CD45+CD11b+F4/80+) in tumour, tumour-draining lymph nodes (TDLN) and spleens from mice receiving indicated treatments. Analysed by one‐way ANOVA with Tukey’s post hoc adjustment (n=5 mice/ condition). Experiment conducted once. *p<0.05, **p<0.01, ***p<0.001. Data presented as mean±SEM. PI3Kγ, phosphoinositide 3-kinase-gamma.

### Macrophage efferocytosis is MERTK dependent

To further explore this mechanism, we measured the expression of MERTK, TYRO3 and AXl, macrophage receptor tyrosine kinases which mediate efferocytosis. We find that MERTK is significantly upregulated on efferocytic macrophages, with the highest levels observed in response to combined treatment, while TYRO3 and AXL are unaffected (figure 6A–C). Blockade of MERTK via a small molecule inhibitor abrogates efferocytosis in PI3Kγ inhibitor treated groups, including irTEM+PI3Kγ inhibition (figure 6D). Changes in expression of additional phagocytic checkpoints were also characterised. The CD24 and SIGLEC-10 axis is largely unchanged, however there is increased expression of CD47 on tumour cells and high levels of SIRPα on macrophages (online supplemental figure 6G–J). Despite this, CD47 blockade did not affect efferocytosis as seen in response to PI3Kγ inhibition (online supplemental figure 6K). These results further confirm that tumour cell engulfment is mediated via efferocytosis and not phagocytosis.

We next tested whether macrophage efferocytosis of tumour cells persisted in vivo by repeating the experiment outlined in figure 1G but with fluorescent KPC-F cells (KPC-F-mCherry). We isolated tumours, tumour-draining lymph nodes and the spleens from treated mice 5 days following the final fraction of radiation. Dissociated tissues were analysed by flow cytometry, quantifying the number of mCherry+ macrophages and dendritic cells (figure 6F). We find significantly more mCherry+ TAMs in tumours and TDLNs of mice receiving combined treatment, which is not seen with conventional dendritic cells (figure 6F; online supplemental figure 6F). To further corroborate the presence of macrophage efferocytosis in vivo, the transcript levels of epithelial, fibroblast and immune cell specific markers in the sorted tumour macrophages populations were counted to determine whether passenger transcripts had been acquired. Very low levels of genes normally found in T cells, fibroblasts and NK cells are detected in TAMs (online supplemental figure 6M). However, epithelial transcripts are markedly elevated in the combination treated groups compared with either control or IR alone (online supplemental figure 6N).

These findings suggest that the unique combination of IR and PI3Kγ inhibition leads to increased efferocytosis by tumour macrophages, a phenomenon that persists in vivo.

These results suggest that macrophage efferocytosis contributes to the antitumor effect in vivo. To assess the extent to which the effect of combined treatment on tumour progression is dependent on macrophages, we performed TAM depletion using αCSF. We observed that αCSF, either alone or in combination with IR, delays tumour growth and extends survival compared with animals treated with the isotype control (figure 6G). In the IR+ PI3Kγ inhibitor group, macrophage depletion abrogates the treatment effect, resulting in tumour growth and survival comparable to the IR+αCSF group (figure 6G; online supplemental figure 6O). Since αCSF independently influenced the progression of both IR and non-IR treated tumours, we sought to specifically target efferocytosis without concurrent macrophage depletion. We find that unlike αCSF, MERTK inhibition does not affect survival in the non-IR and IR groups. In the IR+PI3Kγ group, the treatment effect is abrogated, and survival is the same as animals receiving IR±UNC2250 (figure 6H; online supplemental figure 6P).

To confirm whether efferocytosis is affected by MERTK inhibition, we quantified efferocytic macrophages in tumours, draining lymph nodes and the spleen of treated animals. We find that the previously observed increase in efferocytic macrophages within tumours and draining lymph nodes was reversed following MERTK inhibition (figure 6I). These results suggest that MERTK dependent macrophage efferocytosis is associated with the reduction in tumour growth and increased survival in response to IR+PI3Kγ inhibition.

### Efferocytic macrophages present tumour antigens and prime CD8+ T cells

**Figure 7:**
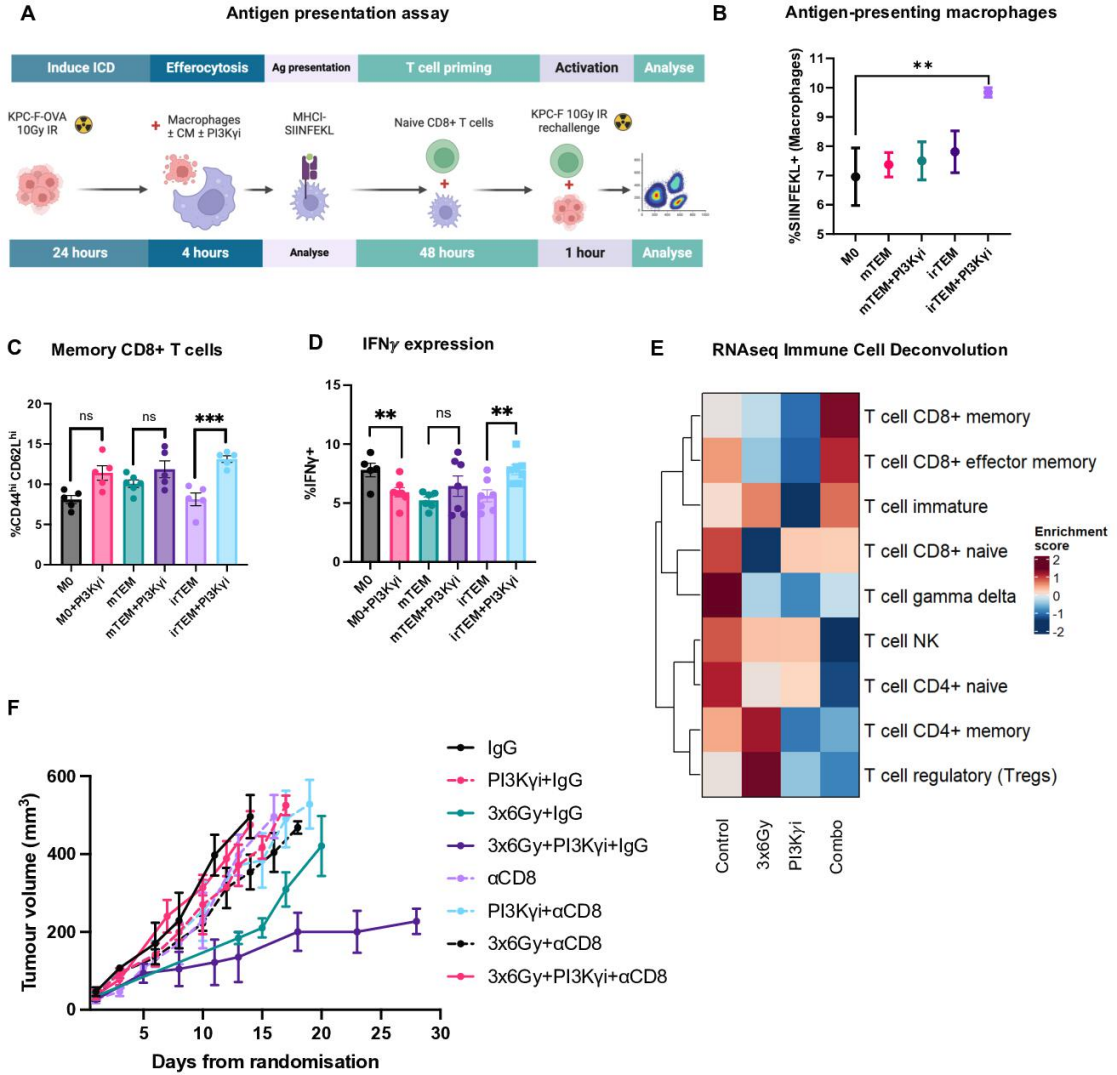
Combined treatment increases antigen presentation locally and distally, increasing the number and potency of effector and memory CD8+ T cells and inducing immunity in vivo. (**A**) Schematic illustrating workflow for antigen presentation and CD8+ T cell activation assay. (**B**) Flow cytometric analysis of SIINFEKL expression on tumour-educated macrophages (TEMs) co-cultured for 4 hours with irradiated KPC-eGFP;OVA. Analysed by one‐way analysis of variance (ANOVA) with Tukey’s post hoc adjustment (n=3). (**C**) Flow cytometric analysis of memory (CD44^hi^CD62L^hi^) CD8+ T cells after co-culture with macrophages (treated as per 7B). Data are presented as mean±SEM and analysed by analysed by one‐way ANOVA with Tukey’s post hoc adjustment (n*≥*5). (**D**) Flow cytometric analysis of IFNγ expression by memory CD8+ T cells. Analysed by one‐way ANOVA with Tukey’s post hoc adjustment (n*≥*5). (**E**) Immune deconvolution, with a focus on T cell phenotypes, of each treatment group performed on bulk tumour RNAseq data from mice obtained in figure 1G. (**F**) Tumour growth kinetics of orthotopic tumours in mice receiving treatments as indicated. Dashed lines correspond with groups receiving concurrent αCD8 antibody. Analysed by one‐way ANOVA with Tukey’s post hoc adjustment (n=5 mice/group). Data are shown from a single experiment. *p<0.05, **p<0.01, ***p<0.001. PI3Kγ, phosphoinositide 3-kinase-gamma.

Analysis of TAMs by RNA sequencing revealed upregulation of antigen processing pathways. Therefore, we sought to determine whether efferocytosis leads to improved tumour antigen presentation. We transfected KPC-F cells to express the model antigen OVALBUMIN and quantified MHCI-SIINFEKL expression on efferocytic macrophages (figure 7A). Combined treated increases the expression of MHCI-SIINFEKL on the surface of macrophages exposed to irTCM+PI3Kγ inhibition, suggesting improved tumour-specific antigen presentation following efferocytosis (figure 7B). We next tested the ability of these efferocytic macrophages to cross-present antigen and activate naïve CD8+ T cells. Efferocytic macrophages stimulates the expansion of CD8+ T cells with a memory phenotype, which express more IFN-γ when re-challenged with irradiated KPC-F cells (figure 7 C,D). Consistent with these findings, immune cell deconvolution of the tumour RNA sequencing data shows that memory CD8+ T cells are most significantly elevated in the combination treatment group (figure 7F). These results further suggest that the phenotypic effect of combined treatment on tumour growth is mediated via adaptive immunity. To confirm, we tested the treatment combinations in mice pretreated with a CD8 depleting antibody, finding that the suppression of tumour growth in the IR+PI3Kγ inhibitor group is abrogated in the absence of CD8+ T cells (figure 7F).

Due to the evidence of increased antigen priming, we tested whether combined treatment increases sensitivity to immune checkpoint blockade. The addition of αPD-1 alongside IR±PI3Kγ inhibition yielded no improvement in outcome, despite an increase in CD8+ T cells in the triple combination group compared with IR+PI3Kγ inhibition alone (online supplemental figure 7A,B). Furthermore, macrophages are significantly reduced in all groups receiving αPD-1, with the most pronounced effect in the triple combination group (online supplemental figure 7C). There was no significant increase in CD8+ T cells in response to anti-PD-1 (online supplemental figure 7D). This unexpected result prompted us to investigate whether PD-1 is expressed on TAMs and whether PD-1 blockade influences viability or induces apoptosis. Analysis of the scRNAseq data revealed low levels of PD-1 expression across all TAM subsets (online supplemental figure 7 E,F). Consistent with these observations, murine TEMs display low levels of PD-1 and do not upregulate its expression in response to PI3Kγ inhibition (online supplemental figure 7G). Interestingly, PD-1 expression was significantly increased in mTEMs treated with PI3Kγ inhibition but not in the irTEM+PI3Kγ inhibitor group (online supplemental figure 7G). Next, we assessed the impact of PD-1 blockade on macrophage efferocytosis and found no differences following treatment (figure 7H). Finally, we examined whether αPD-1 treatment induces apoptosis in macrophages and whether this correlates with PD-1 expression. We observed fewer live cells in the mTEM+PI3Kγ inhibitor group, with a significant increase in apoptotic and necrotic cells (online supplemental figure 7I–K). In line with the low PD-1 expression levels in other groups, apoptosis levels were not significantly altered. These findings suggest that PI3Kγ signalling may influence PD-1 expression in macrophages, and that blocking this pathway via anti-PD-1 induces apoptosis. However, this effect is not consistently observed across groups in vitro and does not fully account for the marked reduction in TAMs observed in vivo. In summary, in a subset of macrophages PI3Kγ signalling modulates PD-1 expression and viability in response to anti-PD-1, though this effect does not fully account for the observed reduction in TAMs in vivo.

## Discussion

Here, we show that PI3Kγ inhibition capitalises on the immune stimulatory effects of IR and uniquely demonstrate that tumour macrophages present tumour antigens via efferocytosis, facilitating adaptive immunity. This overcomes resistance to CD8+ T cell mediated antitumour immunity, reducing tumour growth and extending survival.

Since it was reported that PI3Kγ signalling controls macrophage polarisation, preclinical studies have shown that PI3Kγ inhibition is capable of reversing macrophage-driven immune suppression and improving responses to immune checkpoint blockade.^1 2 20^ We have reported that optimal results are typically seen in the setting of combination therapy, though a minority include radiotherapy, and none do so in pancreatic cancer.^3^ Consistent with these reports, we found that PI3Kγ inhibitor monotherapy only modestly delays tumour growth in pancreatic cancer, while robust effects were seen when combined with IR. Interestingly, we found that IR alone results in macrophage activation with upregulation of pathways that are associated with both immune stimulation and suppression. Despite the potential immune priming effects of IR, it is likely that antitumour activity is thwarted by the simultaneous activation of immune suppressive signals.^11 21 22^ In our model, IR stimulated the release of cytokines known to recruit and polarise myeloid cells (online supplemental figure 4A), which corresponded with both increased number and activation of macrophages (figure 3A, online supplemental figure E–F,I). Indeed, previously published strategies have focused on adjunct depletion of myeloid cells to circumvent these immunosuppressive signals.^21 23 24^ Our study demonstrates that concurrent inhibition of PI3Kγ reprograms tumour macrophages towards an inflammatory antigen-presenting phenotype, without affecting their viability or infiltration within tumours.

In response to combination therapy, CD8+ T cells were more abundant, proliferative and in close contact with tumour cells, suggesting that the effect of combined treatment on tumour growth is CD8+ T cell mediated. Giving credence to this was the abrogation of effect on CD8+ T cell depletion. Importantly, we note that although there were more CD8+ T cells in response to combined therapy, they were still more abundant in untreated tumours. The quantity of CD8+ T cells comprises a single factor among several others that contribute to the quality of antitumour response. A key determinant is antigen specificity, and although dendritic cells are classically described as the archetypal APC, other stromal cells including macrophages and fibroblasts are capable of presenting antigens.^25 26^ Among dendritic cell subtypes, conventional dendritic cell type-1 (cDC1) have been shown to be crucial for driving T cell priming in the tumour setting.^27 28^ However, in established PDAC there are fewer cDC1s, which are dysfunctional and fail to stimulate antitumour immunity.^29 30^

Due to their abundance in many solid tumours, using the antigen-presenting properties of macrophages is appealing. However, although macrophages are known to be capable antigen presenters, this does not necessarily translate into effective T cell activation.^31^ This requires simultaneous engagement of co-stimulatory pathways and an absence of co-inhibitory receptor interactions. Although this phenotype is demonstrated in some specific macrophage subsets, an immunosuppressive phenotype may dominate in TAMs.^31^,^33^ By preventing efferocytosis driven macrophage repolarisation and retaining an inflammatory phenotype, PI3Kγ inhibition may overcome this to both prime and activate CD8+ T cells.

Efferocytosis is typically associated with a shift in macrophages towards an anti-inflammatory, wound-repair phenotype. Consequently, blocking efferocytosis to reverse tumour macrophage polarisation has been explored in preclinical studies.^34^ However, most studies of efferocytosis have focused on treatment-naïve tumours. In this context, efferocytosis has been shown to promote early metastatic growth in the liver by enhancing immunosuppression.^35^ In primary tumours, the clearance of apoptotic tumour cells by macrophages reduces the release of free DNA from necrotic tumour cells, thereby limiting STING activation and its associated antitumour immune effects.^36^ In an analysis of human pancreas tumours, efferocytosis signatures were limited to a single macrophage subpopulation.^37^ Interestingly, antigen processing and presentation pathways were enriched in these macrophages, suggesting that efferocytosis is not a ubiquitous process across tumour macrophage subsets but is likely context-dependent.

Determining the relative contribution of each major class of APC to the overall adaptive immune response is challenging. In comparative analyses of immunotherapy resistant and sensitive tumour models, TAMs were instrumental in the generation of antigen specific antitumour immunity and comparable to DCs in their T cell priming ability.^33^ Additionally, taking advantage of the antigen presenting capabilities of TAMs, investigators have recently demonstrated that targeted antigen delivery to TAMs, as well as chimeric antigen receptor macrophages effectively boosts antitumour immunity.^38 39^ In summary, current evidence highlights dendritic cells as the superior cells for antigen cross-presentation. However, our findings demonstrate that macrophages can be therapeutically manipulated to significantly contribute to antigen presentation and enhance antitumour immunity, particularly in tumours rich in macrophages. Although the increase in efferocytosis following IR+PI3Kγ inhibition in our experiments was modest (from 0.52% to 1.88%), recent studies suggest that even small increases can lead to meaningful antitumor responses and substantial improvements in survival.^30 40^

We detected no additional benefit of anti-PD-1 therapy in our model. Importantly, we unexpectedly saw a marked reduction in the number of tumour macrophages in anti-PD-1 treated groups. While the expression of checkpoint ligands on myeloid cells within the TME has been widely explored, the expression of checkpoint molecules is often neglected. We found evidence that both murine and human colorectal tumour macrophages can express PD-1, with expression levels impacting phagocytic capacity.^41^ However, we find that pancreatic TAMs express PD-1 at very low levels, and anti-PD-1 treatment does not affect efferocytosis or viability. Several studies have reported a reduction in macrophage populations in response to combined anti-PD-1 treatment, although no mechanistic explained is discussed.^220 42^,^44^ Previously, we showed that macrophage depletion delayed tumour growth following radiotherapy in mouse models of colon and pancreas cancer.^45^ In contrast, we observed here that the reduction in macrophages associated with anti-PD-1 does not affect tumour growth or survival. Interestingly, macrophage depletion via anti-CSF delayed tumour growth and improved survival in a manner consistent with our previous findings and reports from other groups.^11 46^ Consistent among these reports is the finding that anti-CSF does not totally deplete TAMs; instead, it leaves a residual population that undergoes repolarisation, which is associated with augmented CD8+ T cell activity. In the context of anti-PD-1, the remaining TAMs may not undergo similar phenotypic changes, potentially explaining the difference in tumour outcomes between anti-CSF- and anti-PD-1-induced TAM reduction.

In patients, radiotherapy is currently being evaluated in the neoadjuvant, intraoperative and adjuvant setting. To date, trial data is conflicting with insufficient evidence to justify the addition of radiotherapy to standard of care chemotherapy.^47 48^ More recently, stereotactic ablative radiotherapy has been evaluated for the treatment of locally advanced pancreatic cancer as well as during the neoadjuvant window.^49^ In the preclinical setting, hypofractionated regimes have been shown to potentiate immunotherapy response in several solid tumour settings. While these approaches have translated into promising clinical trial results for some cancers,^50^ pancreatic cancer remains refractory. Patients with localised pancreatic cancer typically fall into two groups: those with unidentified micrometastases and those without. There is no diagnostic test currently available that can differentiate between these two distinct populations. Based on our preclinical findings, patients with truly localised disease may benefit the most from IR+PI3Kγ inhibition, while those with micrometastases may experience delayed onset of systemic disease.

In summary, we demonstrate that irradiation potentiates the effect of PI3Kγ inhibition on tumour macrophages in pancreatic cancer. Several PI3Kγ inhibitors are in early phase clinical trials and the data presented here suggest therapeutic rationale for combined therapy with IR in patients with localised pancreatic cancer.

## Supplementary material

10.1136/gutjnl-2024-333492 online supplemental file 1

10.1136/gutjnl-2024-333492 online supplemental file 2

10.1136/gutjnl-2024-333492 online supplemental file 3

10.1136/gutjnl-2024-333492 online supplemental file 4

10.1136/gutjnl-2024-333492 online supplemental file 5

10.1136/gutjnl-2024-333492 online supplemental file 6

10.1136/gutjnl-2024-333492 online supplemental file 7

10.1136/gutjnl-2024-333492 online supplemental file 8

## Data Availability

Data are available upon reasonable request.
