# Supplementary material for: Induction of macrophage efferocytosis in pancreatic cancer via PI3Kγ inhibition and radiotherapy promotes tumour control
Source: Gut. 2025 Jan 9;74(5):e333492. doi: 10.1136/gutjnl-2024-333492 (PMC12013568; doi:10.1136/gutjnl-2024-333492)
Supplement: online supplemental file 1 [file gutjnl-74-5-s001.pdf]

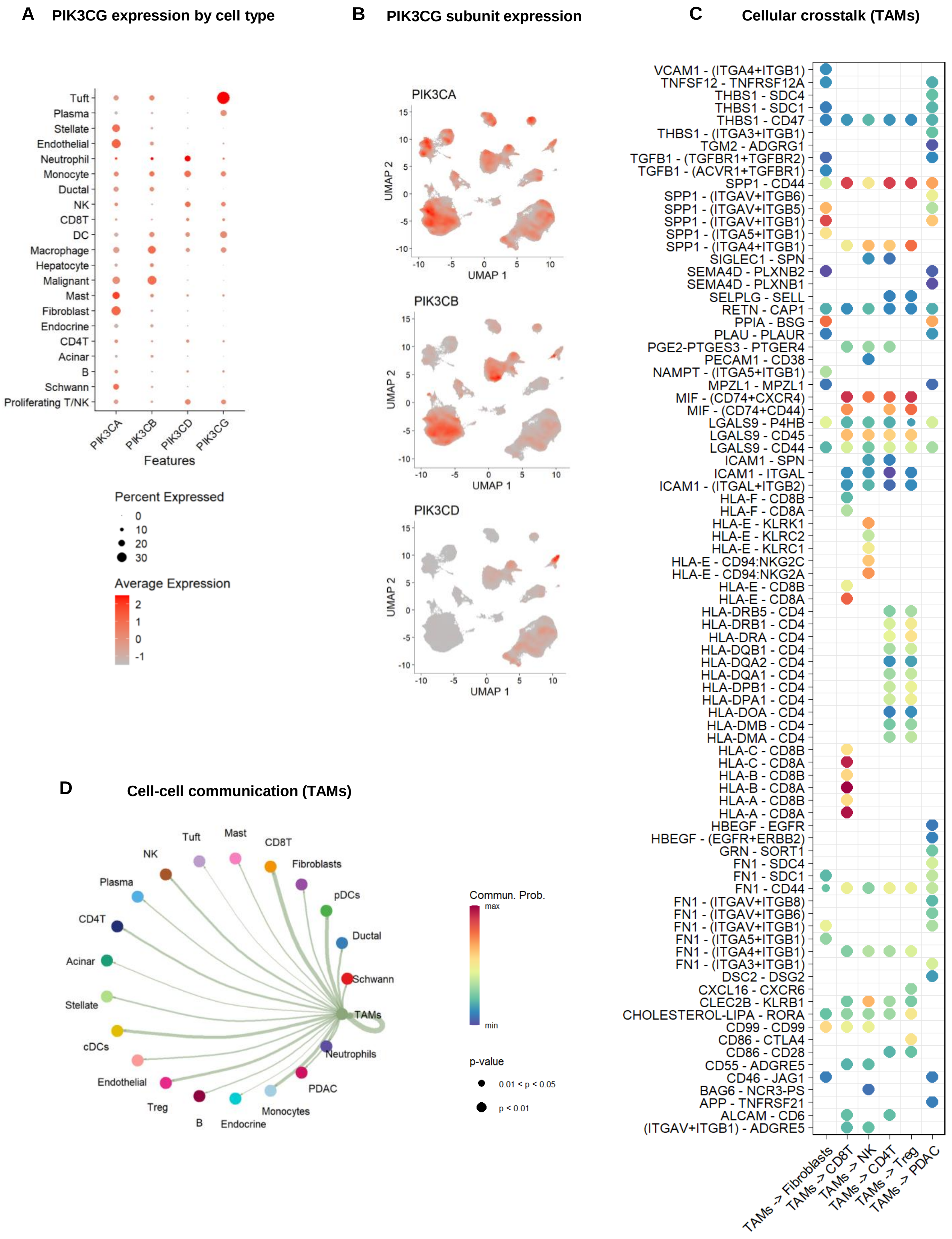
A
PIK3CG expression by cell type
Tuft
Plasma
Stellate
Endothelial
Neutrophil
Monocyte
Ductal
NK
CD8T
DC
Macrophage
Hepatocyte
Malignant
Mast
Fibroblast
Endocrine
CD4T
Acinar
B
Schwann
Proliferating T/NK
PIK3CA
PIK3CB
PIK3CD
PIK3CG
Features
Percent Expressed
0
10
20
30
Average Expression
2
1
0
-1
B
PIK3CG subunit expression
PIK3CA
PIK3CB
PIK3CD
UMAP 1
UMAP 2
C
Cellular crosstalk (TAMs)
VCAM1 - (ITGA4+ITGB1)
TNFSF12 - TNFRSF12A
THBS1 - SDC4
THBS1 - SDC1
THBS1 - CD47
THBS1 - (ITGA3+ITGB1)
TGM2 - ADGRG1
TGFB1 - (TGFBR1+TGFBR2)
TGFB1 - (ACVR1+TGFBR1)
SPP1 - CD44
SPP1 - (ITGAV+ITGB6)
SPP1 - (ITGAV+ITGB5)
SPP1 - (ITGAV+ITGB1)
SPP1 - (ITGA5+ITGB1)
SPP1 - (ITGA4+ITGB1)
SIGLEC1 - SPN
SEMA4D - PLXNB2
SEMA4D - PLXNB1
SELPLG - SELL
RETN - CAP1
PPIA - BSG
PLAU - PLAUR
PGE2-PTGES3 - PTGER4
PECAM1 - CD38
NAMPT - (ITGA5+ITGB1)
MPZL1 - MPZL1
MIF - (CD74+CXCR4)
MIF - (CD74+CD44)
LGALS9 - P4HB
LGALS9 - CD45
LGALS9 - CD44
ICAM1 - SPN
ICAM1 - ITGAL
ICAM1 - (ITGAL+ITGB2)
HLA-F - CD8B
HLA-F - CD8A
HLA-E - KLRK1
HLA-E - KLRC2
HLA-E - KLRC1
HLA-E - CD94:NKG2C
HLA-E - CD94:NKG2A
HLA-E - CD8B
HLA-E - CD8A
HLA-DRB5 - CD4
HLA-DRB1 - CD4
HLA-DRA - CD4
HLA-DQB1 - CD4
HLA-DQA2 - CD4
HLA-DQA1 - CD4
HLA-DPB1 - CD4
HLA-DPA1 - CD4
HLA-DOA - CD4
HLA-DMB - CD4
HLA-DMA - CD4
HLA-C - CD8B
HLA-C - CD8A
HLA-B - CD8B
HLA-B - CD8A
HLA-A - CD8B
HLA-A - CD8A
HBEGF - EGFR
HBEGF - (EGFR+ERBB2)
GRN - SORT1
FN1 - SDC4
FN1 - SDC1
FN1 - CD44
FN1 - (ITGAV+ITGB8)
FN1 - (ITGAV+ITGB6)
FN1 - (ITGAV+ITGB1)
FN1 - (ITGA5+ITGB1)
FN1 - (ITGA4+ITGB1)
FN1 - (ITGA3+ITGB1)
DSC2 - DSG2
CXCL16 - CXCR6
CLEC2B - KLRB1
CHOLESTEROL-LIPA - RORA
CD99 - CD99
CD86 - CTLA4
CD86 - CD28
CD55 - ADGRE5
CD46 - JAG1
BAG6 - NCR3-PS
APP - TNFRSF21
ALCAM - CD6
(ITGAV+ITGB1) - ADGRE5
TAMs -> Fibroblasts
TAMs -> CD8T
TAMs -> NK
TAMs -> CD4T
TAMs -> Treg
TAMs -> PDAC
Commun. Prob.
max
min
p-value
0.01 < p < 0.05
p < 0.01
D
Cell-cell communication (TAMs)
Tuft
Mast
CD8T
NK
Fibroblasts
Plasma
pDCs
CD4T
Ductal
Acinar
Schwann
Stellate
TAMs
cDCs
Neutrophils
Endothelial
PDAC
Treg
B
Endocrine
Monocytes

Supplementary Figure 1: Analysis of human PDAC scRNAseq data regarding expression of PI3K isoforms and cellular crosstalk.

(A) Bubble plot representing expression of different PI3K subunits across cellular compartments.

(B) Expression of PI3Kα, PI3Kβ and PI3Kδ subunits across different broad cell clusters as depicted in Figure 1A.

(C) Cellular crosstalk network between tumour-associated macrophages (TAMs) and other cell types as identified in scRNAseq analysis.

(D) Cell-cell communication between tumour macrophages and other cells in the tumour microenvironment. The thickness of the connecting line indicates the strength of association.
