# Supplementary material for: Induction of macrophage efferocytosis in pancreatic cancer via PI3Kγ inhibition and radiotherapy promotes tumour control
Source: Gut. 2025 Jan 9;74(5):e333492. doi: 10.1136/gutjnl-2024-333492 (PMC12013568; doi:10.1136/gutjnl-2024-333492)
Supplement: online supplemental file 2 [file gutjnl-74-5-s002.pdf]

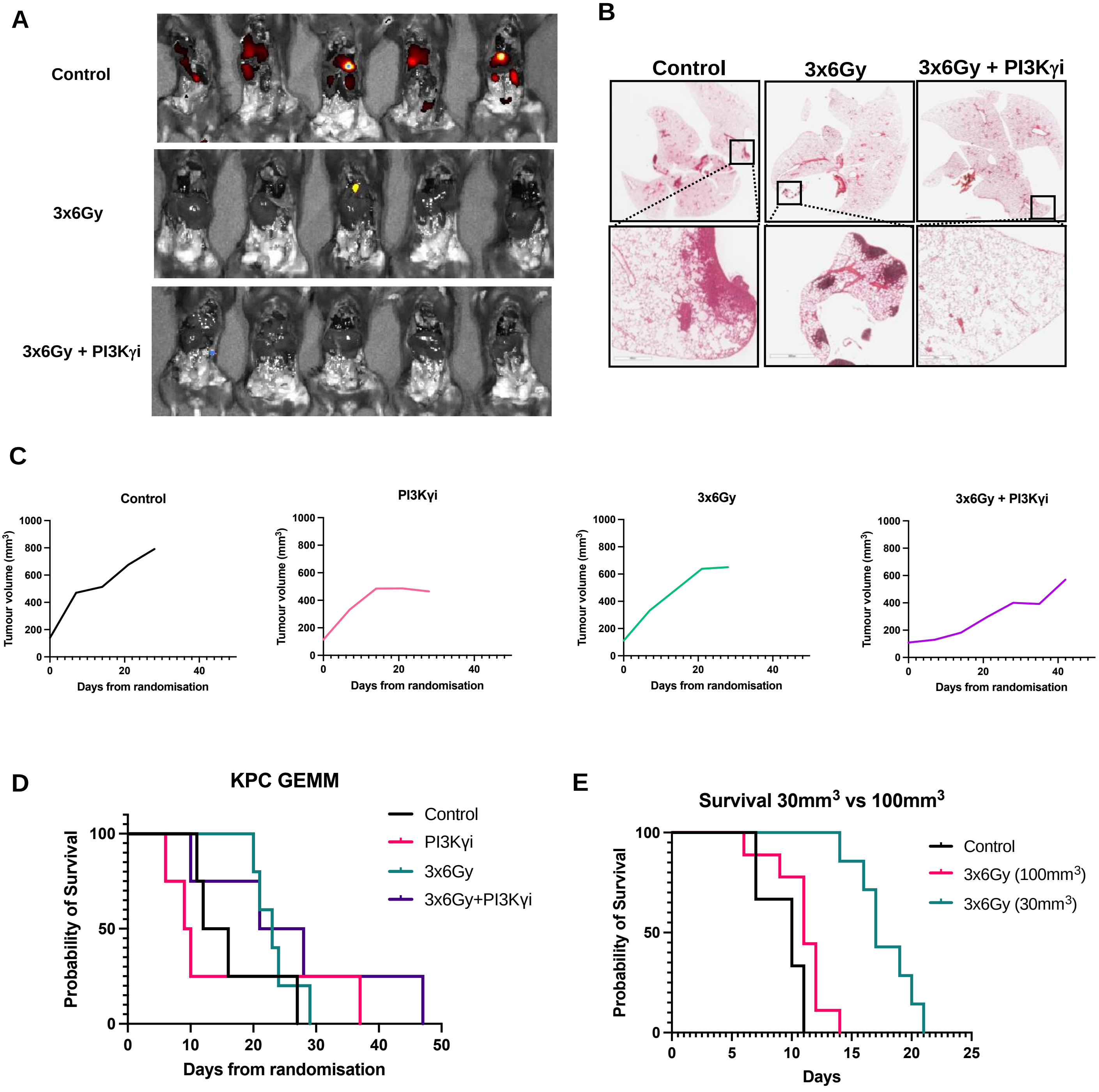
A
Control
3x6Gy
3x6Gy + PI3Kγi
B
Control
3x6Gy
3x6Gy + PI3Kγi
C
Control
PI3Kγi
3x6Gy
3x6Gy + PI3Kγi
Tumour volume (mm3)
Days from randomisation
D
KPC GEMM
Probability of Survival
Days from randomisation
Control
PI3Kγi
3x6Gy
3x6Gy+PI3Kγi
E
Survival 30mm3 vs 100mm3
Probability of Survival
Days
Control
3x6Gy (100mm3)
3x6Gy (30mm3)

Supplementary Figure 2: Analysis of metastatic burden in orthotopic and GEMM pancreatic cancer models.

(A) Bioluminescence images of mice to determine metastatic burden. Mice bearing orthotopic KPC-F-mCherry tumours were treated with IR±PI3Kγ inhibitor were culled and primary pancreatic tumours resected via laparotomy. Mice were then imaged using the IVIS system to detect fluorescent signal at 550-650nm.

(B) Representative H+E images of lungs isolated from mice receiving treatment as indicated. Mice were culled 11 days following randomisation (5 days following the final fraction of IR) and lungs isolated, infused with agarose and fixed with Bouin's solution.

(C) Tumour growth kinetics of GEM KPC mice with confirmed primary PDAC tumours as treated in Figure 1G. Data are presented as mean tumour volume ± SEM and analysed by one-way ANOVA with Tukey's *post hoc* adjustment (*n* = 6 mice/group). Experiment conducted once.

(D) Kaplan–Meier survival curve of KPC treated from detection of palpable tumour, confirmed by ultrasound. Experiment conducted once.

(E) Kaplan-Meier survival curve of mice bearing orthotopic KPC tumours receiving 3x6Gy irradiation. The difference between treatment groups is the tumour volume at the point of randomisation and treatment initiation (30mm^3 vs 100mm^3).
