# Supplementary material for: Induction of macrophage efferocytosis in pancreatic cancer via PI3Kγ inhibition and radiotherapy promotes tumour control
Source: Gut. 2025 Jan 9;74(5):e333492. doi: 10.1136/gutjnl-2024-333492 (PMC12013568; doi:10.1136/gutjnl-2024-333492)
Supplement: online supplemental file 3 [file gutjnl-74-5-s003.pdf]

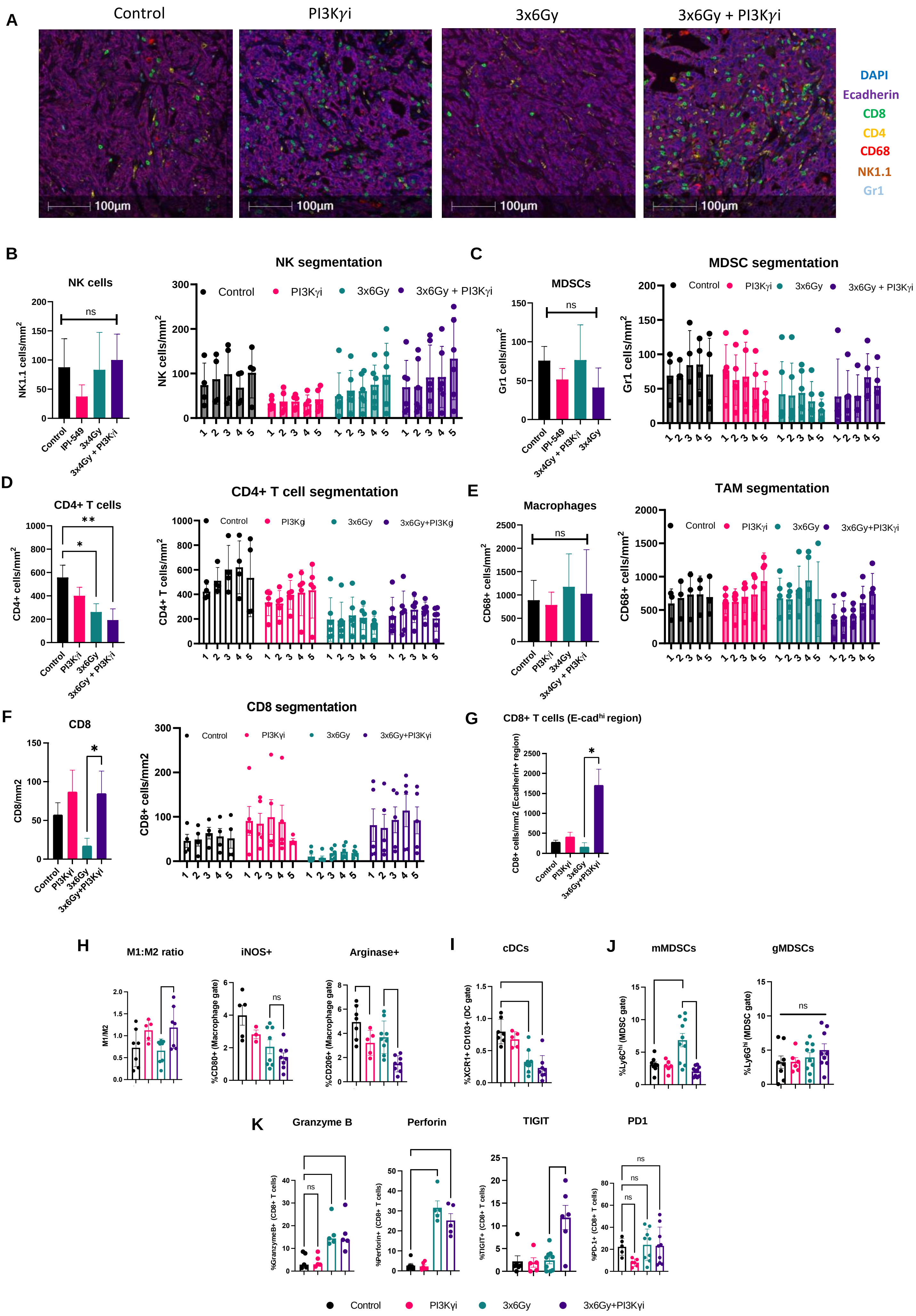
A
Control
PI3Kγi
3x6Gy
3x6Gy + PI3Kγi
100µm
DAPI
Ecadherin
CD8
CD4
CD68
NK1.1
Gr1
B
NK cells
NK segmentation
NK1.1 cells/mm2
NK cells/mm2
ns
Control
IPI-549
3x4Gy
3x4Gy + PI3Kγi
C
MDSCs
MDSC segmentation
Gr1 cells/mm2
D
CD4+ T cells
CD4+ T cell segmentation
CD4+ cells/mm2
CD4+ T cells/mm2
E
Macrophages
TAM segmentation
CD68+ cells/mm2
F
CD8
CD8 segmentation
CD8/mm2
CD8+ cells/mm2
G
CD8+ T cells (E-cadhi region)
CD8+ cells/mm2 (Ecadherin+ region)
H
M1:M2 ratio
iNOS+
Arginase+
M1/M2
%CD80+ (Macrophage gate)
%CD206+ (Macrophage gate)
I
cDCs
%XCR1+ CD103+ (DC gate)
J
mMDSCs
gMDSCs
%Ly6Chi (MDSC gate)
%Ly6Ghi (MDSC gate)
K
Granzyme B
Perforin
TIGIT
PD1
%GranzymeB+ (CD8+ T cells)
%Perforin+ (CD8+ T cells)
%TIGIT+ (CD8+ T cells)
%PD-1+ (CD8+ T cells)
Control
PI3Kγi
3x6Gy
3x6Gy+PI3Kγi

Supplementary Figure 3: Immunofluorescent analysis of cell types across treated tumour samples.

(A) Representative sections of tumours from mice receiving treatments as indicated and stained with a multiplex immunofluorescence panel. Blue = DAPI, purple = E-cadherin, green = CD8, blue = CD4, red = CD68, orange = NK1.1, Cyan = Gr1. Sections presented in panel A are identical to those in Figure 3D but with all fluorescent channels and markers displayed.

(B-F) (B) NK, (C) MDSC, (D) CD4+ T cells, (E) TAMs and (F) CD8+ T cells were quantified in serial segments of tumour slices from the centre to the periphery (1-5) across each treatment group. Left panels illustrate total cells found per mm^2 of tumour, whilst right panels show the change in cell numbers within each treatment group. Data are presented as mean ± SEM and analysed by one-way ANOVA with Tukey's *post hoc* adjustment ($n = 5$ mice/ group).

(G) Quantification of the number of CD8+ T cells in E-cad^{hi} regions of the tumour. Data are presented as mean ± SEM and analysed by two-tailed, one sample t-tests.

(H) Flow cytometric analysis of M1 TAMs (CD11b+ F4/80+ CD80+) and M2 TAMs (CD11b+ F4/80+ CD206+) and the M1:M2 ratio. Analysed by one-way ANOVA with Tukey's *post hoc* adjustment (n = 5-9/group). Data representative of flow cytometry analysis of tumours from three independent experiments.

(I) Flow cytometric analysis of conventional type 1 dendritic cells in dissociated tumours from mice receiving indicated treatments (CD45+ CD11b- F4/80- CD11c+ XCR1+ CD103+ MHCII+). Analysed by one-way ANOVA with Tukey's *post hoc* adjustment (n = 5-9/group). Data representative of flow cytometry analysis of tumours from three independent experiments.

(J) Flow cytometric analysis of monocytic MDSCs ($Ly6C^{hi}$ $Ly6G^{lo}$) and granulocytic MDSCs ($Ly6C^{lo}$ $Ly6G^{hi}$) in dissociated tumours from mice receiving treatments as indicated. Analysed by one-way ANOVA with Tukey's *post hoc* adjustment (n = 5-9/group). Data representative of flow cytometry analysis of tumours from three independent experiments.

(K) Flow cytometric analysis of granzyme B, perforin, TIGIT and PD-1 expression on gated CD8+ T cells from dissociated tumours from mice receiving treatments as indicated. Analysed by one-way ANOVA with Tukey's *post hoc* adjustment (n = 5-9/group). Data representative of flow cytometry analysis of tumours from three independent experiments.

$^{*}P < 0.05$, $^{**}P < 0.01$, $^{***}P < 0.001$.
