# Supplementary material for: Induction of macrophage efferocytosis in pancreatic cancer via PI3Kγ inhibition and radiotherapy promotes tumour control
Source: Gut. 2025 Jan 9;74(5):e333492. doi: 10.1136/gutjnl-2024-333492 (PMC12013568; doi:10.1136/gutjnl-2024-333492)
Supplement: online supplemental file 4 [file gutjnl-74-5-s004.pdf]

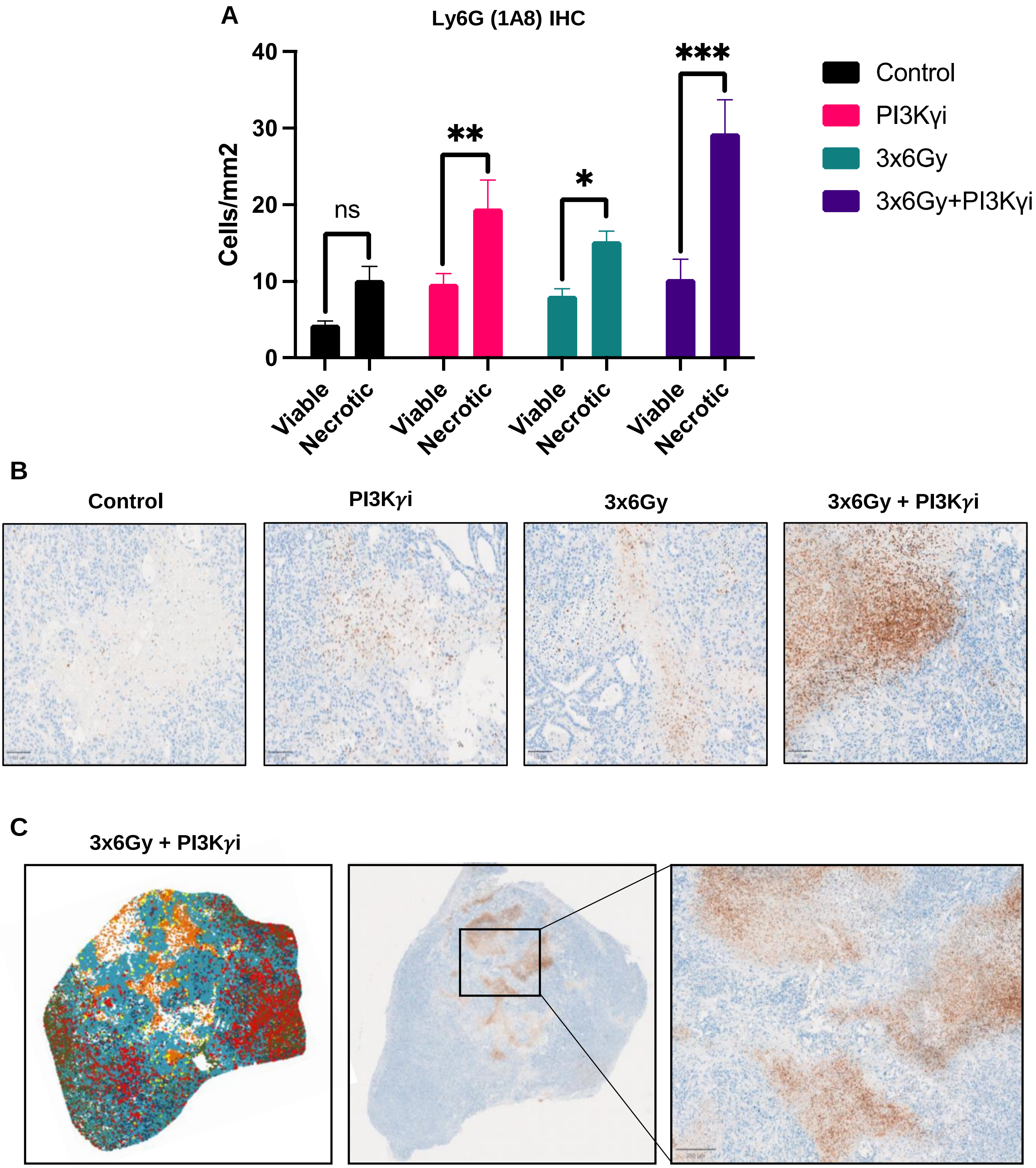

Supplementary Figure 4: Ly6G^{hi} granulocytes infiltrate necrotic areas of KPC-F tumours.

(A) KPC-F tumours sections were stained with anti-Ly6G(1A8) and cell density was quantified and separated into normal and necrotic areas. Data are presented as mean ± SEM and analysed by student's unpaired t-test (n = 5).

(B) Representative immunohistochemistry images of KPC-F tumour sections stained with anti-Ly6G(1A8).

(C) Identical KPC-F tumour sections from the IR+PI3Kγ inhibitor group were stained by immunohistochemistry to detect Ly6G(1A8). Representative image previously shown in Figure 2E is included here.
