# Supplementary material for: Induction of macrophage efferocytosis in pancreatic cancer via PI3Kγ inhibition and radiotherapy promotes tumour control
Source: Gut. 2025 Jan 9;74(5):e333492. doi: 10.1136/gutjnl-2024-333492 (PMC12013568; doi:10.1136/gutjnl-2024-333492)
Supplement: online supplemental file 5 [file gutjnl-74-5-s005.pdf]

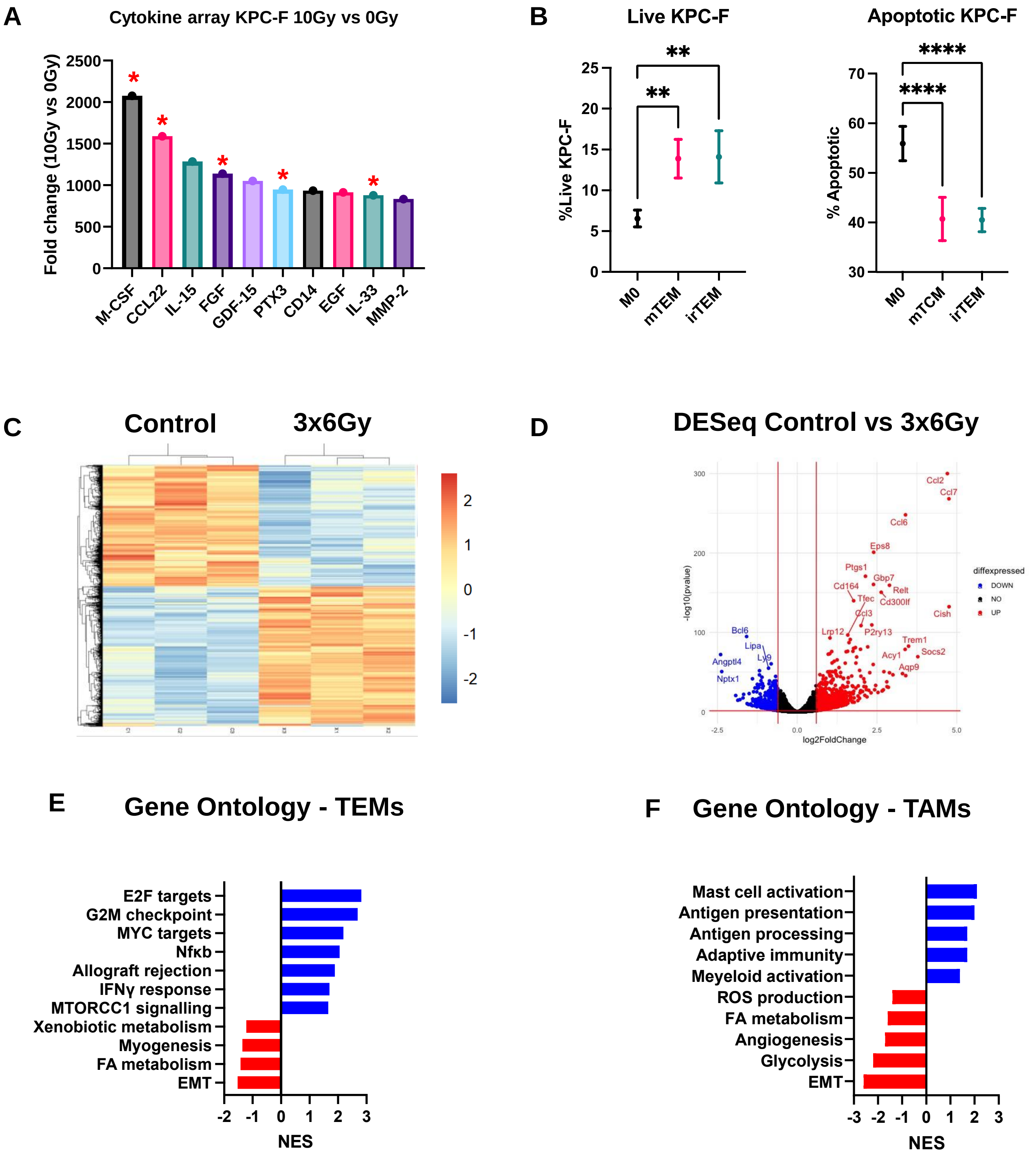

A
Cytokine array KPC-F 10Gy vs 0Gy
Fold change (10Gy vs 0Gy)
2500
2000
1500
1000
500
0
M-CSF
CCL22
IL-15
FGF
GDF-15
PTX3
CD14
EGF
IL-33
MMP-2
B
Live KPC-F
%Live KPC-F
25
20
15
10
5
0
**
**
M0
mTEM
irTEM
Apoptotic KPC-F
% Apoptotic
70
60
50
40
30
****
****
M0
mTCM
irTEM
C
Control
3x6Gy
2
1
0
-1
-2
D
DESeq Control vs 3x6Gy
-log10(pvalue)
300
200
100
0
log2FoldChange
-2.5
0.0
2.5
5.0
Ccl2
Ccl7
Ccl6
Eps8
Ptgs1
Gbp7
Cd164
Relt
Tfec
Cd300lf
Ccl3
Cish
Bcl6
Lrp12
P2ry13
Trem1
Lipa
Acy1
Socs2
Angptl4
Ly9
Aqp9
Nptx1
diffexpressed
DOWN
NO
UP
E
Gene Ontology - TEMs
E2F targets
G2M checkpoint
MYC targets
Nfκb
Allograft rejection
IFNγ response
MTORCC1 signalling
Xenobiotic metabolism
Myogenesis
FA metabolism
EMT
-2
-1
0
1
2
3
NES
F
Gene Ontology - TAMs
Mast cell activation
Antigen presentation
Antigen processing
Adaptive immunity
Meyeloid activation
ROS production
FA metabolism
Angiogenesis
Glycolysis
EMT
-3
-2
-1
0
1
2
3
NES

Supplementary Figure 5: Analysis of changes in cytokine signalling, and subsequent alterations to viability, transcriptomic and migration patterns of immune cells in response to irradiation of KPC-F cells.

(A) Cytokine array demonstrating altered cytokines in the conditioned media of KPC-F cells following 10Gy irradiation calculated by relative fold change. Data is presented as average fold change relative to non-irradiated

(B) Flow cytometric analysis of live or apoptotic KPC-F following a co-culture with TEMs and CD8+ T cells. Data is presented as mean ± SEM and analysed by one-way ANOVA with Tukey's *post hoc* adjustment (n = 3 mice/ group).

(C) RNA sequencing data displaying differentially expressed genes between untreated TEMs versus TEMs exposed to irTCM. A histogram of the log^2fold change of these genes in control and irTEM is shown (n = 3 per condition).

(D) Volcano plot comparing transcriptomic changes between untreated TEMs and irTEMs. Coloured dots represent significantly downregulated (blue) and upregulated (red) genes.

(E) Gene ontology analysis of top upregulated and downregulated pathways between untreated and irTEMs. Pathways significant at $p<0.05$ are presented.

(F) Gene ontology analysis of top upregulated and downregulated pathways comparing CD11b+ cells isolated from tumours in control mice versus mice receiving 3x6Gy+PI3Kγ inhibition. Pathways significant at $p<0.05$ are presented.

*P < 0.05, **P < 0.01, ***P < 0.001.
