# Supplementary material for: Induction of macrophage efferocytosis in pancreatic cancer via PI3Kγ inhibition and radiotherapy promotes tumour control
Source: Gut. 2025 Jan 9;74(5):e333492. doi: 10.1136/gutjnl-2024-333492 (PMC12013568; doi:10.1136/gutjnl-2024-333492)
Supplement: online supplemental file 6 [file gutjnl-74-5-s006.pdf]

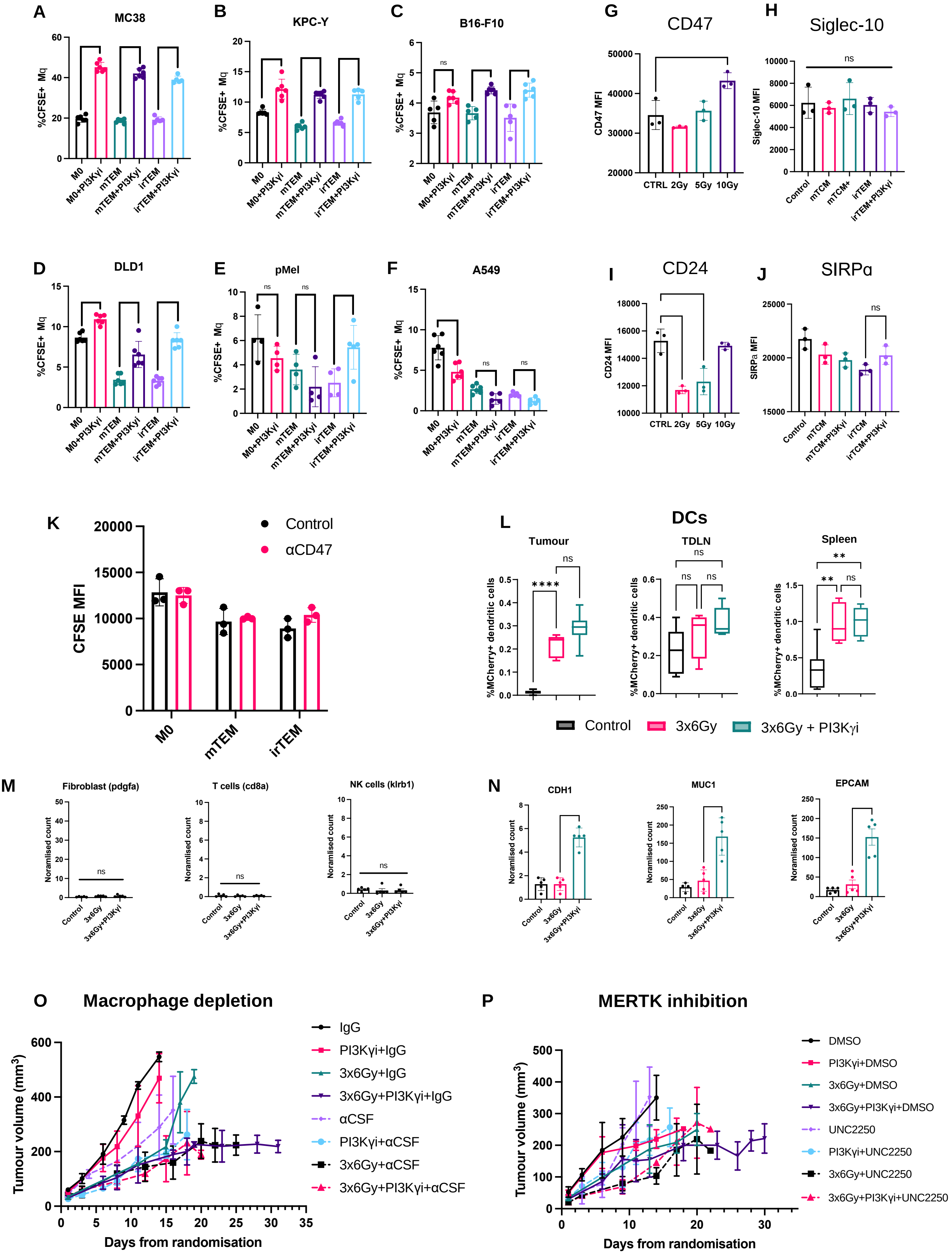

A
MC38
%CFSE+ Mq
M0
M0+PI3Kγi
mTEM
mTEM+PI3Kγi
irTEM
irTEM+PI3Kγi
B
KPC-Y
C
B16-F10
ns
G
CD47
CD47 MFI
CTRL
2Gy
5Gy
10Gy
H
Siglec-10
Siglec-10 MFI
ns
Control
mTCM
mTCM+
irTEM
irTEM+PI3Kγi
D
DLD1
E
pMel
F
A549
I
CD24
CD24 MFI
J
SIRPα
SIRPα MFI
mTCM+PI3Kγi
irTCM
irTCM+PI3Kγi
K
Control
αCD47
CFSE MFI
M0
mTEM
irTEM
L
DCs
Tumour
TDLN
Spleen
%MCherry+ dendritic cells
****
**
Control
3x6Gy
3x6Gy + PI3Kγi
M
Fibroblast (pdgfa)
T cells (cd8a)
NK cells (klrb1)
Noramlised count
3x6Gy+PI3Kγi
N
CDH1
MUC1
EPCAM
O
Macrophage depletion
Tumour volume (mm³)
Days from randomisation
IgG
PI3Kγi+IgG
3x6Gy+IgG
3x6Gy+PI3Kγi+IgG
αCSF
PI3Kγi+αCSF
3x6Gy+αCSF
3x6Gy+PI3Kγi+αCSF
P
MERTK inhibition
DMSO
PI3Kγi+DMSO
3x6Gy+DMSO
3x6Gy+PI3Kγi+DMSO
UNC2250
PI3Kγi+UNC2250
3x6Gy+UNC2250
3x6Gy+PI3Kγi+UNC2250

Supplementary Figure 6: Induction of efferocytosis by IR+PI3Kγ inhibition is not restricted to pancreatic cancer and is dependent on MERTK signalling.

(A-F) Quantification of efferocytosis in macrophages receiving treatment as indicated. Tumour conditioned media was collected from each of the indicated cell lines. Efferocytic macrophages were quantified by measuring total CFSE+ macrophages compared to CFSE- macrophages. Data is presented as mean ± SEM and analysed by one-way ANOVA with Tukey's *post hoc* adjustment (n = 3).

(G) Flow cytometric analysis of CD47 expression in KPC-F tumour cells treated with different doses of radiation. Data is presented as mean ± SEM and analysed by one-way ANOVA with Tukey's *post hoc* adjustment (n = 3).

(H) Flow cytometric analysis of SIGLEC-10 expression in bone marrow-derived macrophages treated with mTCM or irTCM ± PI3Kγ inhibitor. Data is presented as mean ± SEM and analysed by one-way ANOVA with Tukey's *post hoc* adjustment (n = 3).

(I) Flow cytometric analysis of CD24 expression in KPC-F tumour cells treated with different doses of radiation. Data is presented as mean ± SEM and analysed by one-way ANOVA with Tukey's *post hoc* adjustment (n = 3).

(J) Flow cytometric analysis of SIRPα expression in bone marrow-derived macrophages treated with mTCM or irTCM ± PI3Kγ inhibitor. Data is presented as mean ± SEM and analysed by one-way ANOVA with Tukey's *post hoc* adjustment (n = 3).

(K) Quantification of efferocytosis in macrophages receiving treatment as indicated. All groups received αCD47 blocking antibody for the duration of the experiment. Efferocytic macrophages were quantified by measuring total CFSE+ macrophages compared to CFSE- macrophages (as per Figure 5D). Data is presented as mean ± SEM and analysed by one-way ANOVA with Tukey's *post hoc* adjustment (n = 3).

(L) Flow cytometric analysis of mCherry+ dendritic cellsin tumours, tumour-draining lymph nodes (TDLN) and spleens of mice receiving indicated treatments. Analysed by one-way ANOVA with Tukey's *post hoc* adjustment (n = 5 mice/ condition).

(M-N) RNA sequencing was performed on CD11b+ cells isolated from mouse tumours receiving treatments as indicated. Transcript numbers for indicated genes were quantified and compared between groups. Data are presented as mean ± SEM and analysed by analysed by one-way ANOVA with Tukey's *post hoc* adjustment (n = 5).

(O) Tumour growth kinetics of mice bearing orthotopic KPC tumours receiving treatments as indicated. Analysed by one-way ANOVA with Tukey's *post hoc* adjustment (n = 5-6 mice/group). Experiment conducted once.

(P) Tumour growth kinetics of mice bearing orthotopic KPC tumours receiving treatments as indicated. Analysed by one-way ANOVA with Tukey's *post hoc* adjustment (n = 5-6 mice/group). Experiment conducted once.

$^{*}P < 0.05$, $^{**}P < 0.01$, $^{***}P < 0.001$.
