# Supplementary material for: Induction of macrophage efferocytosis in pancreatic cancer via PI3Kγ inhibition and radiotherapy promotes tumour control
Source: Gut. 2025 Jan 9;74(5):e333492. doi: 10.1136/gutjnl-2024-333492 (PMC12013568; doi:10.1136/gutjnl-2024-333492)
Supplement: online supplemental file 7 [file gutjnl-74-5-s007.pdf]

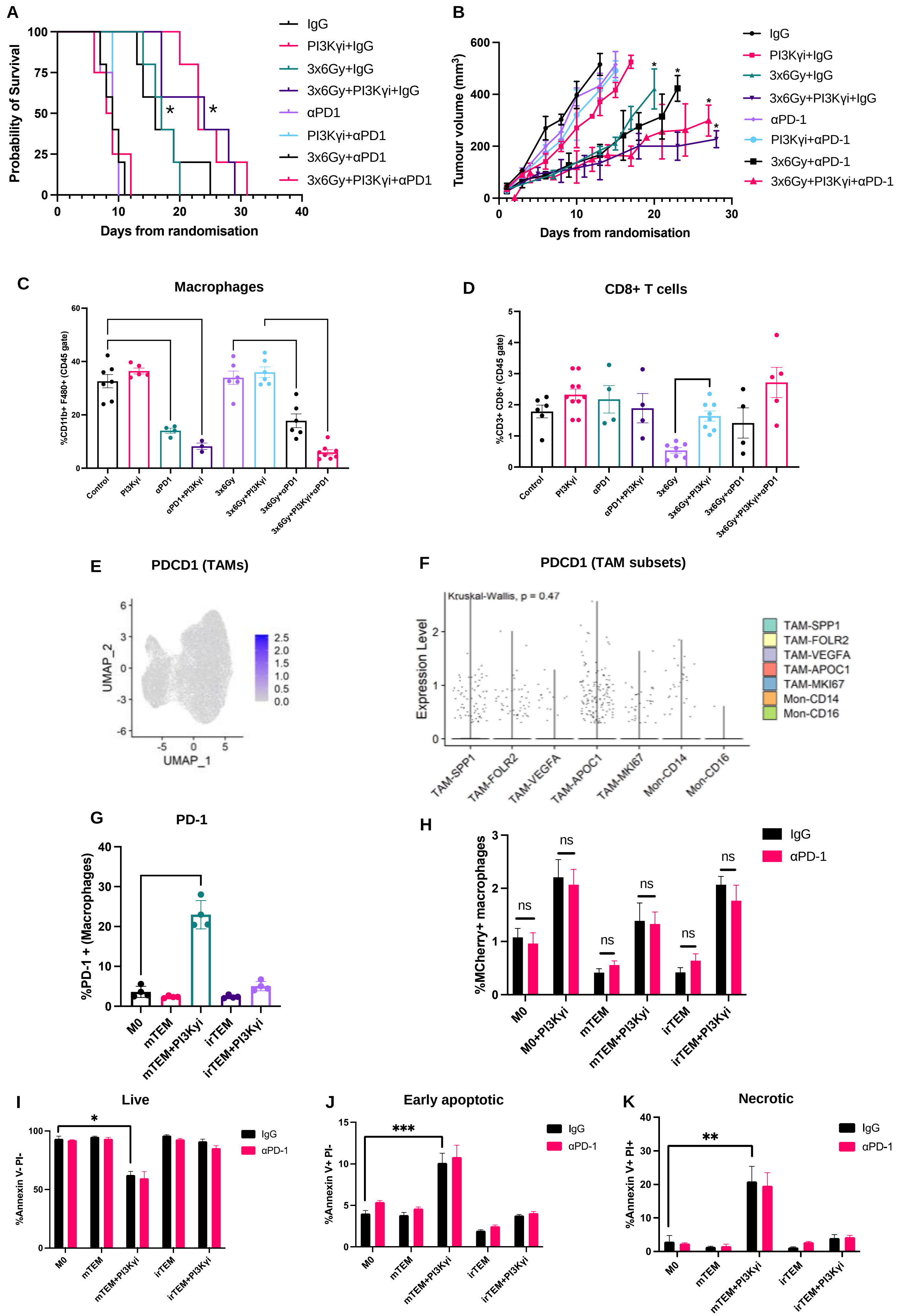

A
Probability of Survival
Days from randomisation
IgG
PI3Kγi+IgG
3x6Gy+IgG
3x6Gy+PI3Kγi+IgG
αPD1
PI3Kγi+αPD1
3x6Gy+αPD1
3x6Gy+PI3Kγi+αPD1
B
Tumour volume (mm^3)
Days from randomisation
IgG
PI3Kγi+IgG
3x6Gy+IgG
3x6Gy+PI3Kγi+IgG
αPD-1
PI3Kγi+αPD-1
3x6Gy+αPD-1
3x6Gy+PI3Kγi+αPD-1
C
Macrophages
%CD11b+ F480+ (CD45 gate)
Control
PI3Kγi
αPD1
αPD1+PI3Kγi
3x6Gy
3x6Gy+PI3Kγi
3x6Gy+αPD1
3x6Gy+PI3Kγi+αPD1
D
CD8+ T cells
%CD3+ CD8+ (CD45 gate)
Control
PI3Kγi
αPD1
αPD1+PI3Kγi
3x6Gy
3x6Gy+PI3Kγi
3x6Gy+αPD1
3x6Gy+PI3Kγi+αPD1
E
PDCD1 (TAMs)
UMAP_2
UMAP_1
F
PDCD1 (TAM subsets)
Kruskal-Wallis, p = 0.47
Expression Level
TAM-SPP1
TAM-FOLR2
TAM-VEGFA
TAM-APOC1
TAM-MKI67
Mon-CD14
Mon-CD16
G
PD-1
%PD-1 + (Macrophages)
M0
mTEM
mTEM+PI3Kγi
irTEM
irTEM+PI3Kγi
H
%MCherry+ macrophages
ns
IgG
αPD-1
M0
M0+PI3Kγi
mTEM
mTEM+PI3Kγi
irTEM
irTEM+PI3Kγi
I
Live
%Annexin V- PI-
IgG
αPD-1
M0
mTEM
mTEM+PI3Kγi
irTEM
irTEM+PI3Kγi
J
Early apoptotic
%Annexin V+ PI-
IgG
αPD-1
K
Necrotic
%Annexin V+ PI+
IgG
αPD-1

Supplementary Figure 7: Immune checkpoint inhibition does not improve survival in response to IR+ PI3Kγ inhibition.

(A) Kaplan–Meier survival plot of mice bearing orthotopic KPC tumours as treated in G ± anti-PD1; log-rank Mantel-Cox test ($p<0.05$)**.** Experiment conducted once.

(B) Tumour growth kinetics of mice bearing orthotopic KPC tumours as treated in G ± anti-PD1. Analysed by one-way ANOVA with Tukey's *post hoc* adjustment (n = 5-6 mice/group). Experiment conducted once.

(C-D) Flow cytometric analysis of macrophages (C: CD45+ CD11b+ F4/80+) and CD8+ T cells (D: CD34+ CD3+ CD8+) in KPC-F tumours receiving indicated treatments. Analysed by one-way ANOVA with Tukey's *post hoc* adjustment (n = 5-9/group). Representative example of flow cytometry analysis of tumours from a single animal experiment.

(E) UMAP derived from human scRNAseq analysis illustrating PDCD1 (PD-1) expression in the pan macrophage cluster.

(F) Expression level of PDCD1 (PD-1) across different TAM subsets derived from human scRNAseq analysis. Analysed by Kruskal-Wallis.

(G) Quantification of surface PD-1 expression on macrophages by flow cytometry. Analysed by one-way ANOVA with Tukey's *post hoc* adjustment.

(H) Flow cytometric analysis of TEMs co-cultured with irradiated KPC-F as per Figure 5D ± UNC2250 to inhibit MERTK. Representative flow cytometry plots are shown. Efferocytic cells were quantified by measuring total CFSE+ cells compared to CFSE- cells. Analysed by two-tailed, one sample t-tests (n = 4-5).

(I-J) Quantification of apoptosis by Annexin V / Propidium Iodide staining in macrophages receiving the indicated treatments. Comparisons within groups between IgG and anti-PD-1 were analysed by two-tailed, one sample t-tests (n = 5).

*P < 0.05, **P < 0.01, ***P < 0.001.
