# Supplementary material for: Induction of macrophage efferocytosis in pancreatic cancer via PI3Kγ inhibition and radiotherapy promotes tumour control
Source: Gut. 2025 Jan 9;74(5):e333492. doi: 10.1136/gutjnl-2024-333492 (PMC12013568; doi:10.1136/gutjnl-2024-333492)
Supplement: online supplemental file 8 [file gutjnl-74-5-s008.pdf]

Materials and Methods

Cell lines and tissue culture

The following cell lines were used in this study:

**Cell line**	**Source**
A549	Gift from Dr Ahmet Hazini, University of Oxford
B16F10	Gift from Dr Hala Estephan, University of Oxford
B3Z	Gift from Dr Ahmet Hazini, University of Oxford
DC2.4	Gift from Dr Giampiero Valenzano, University of Oxford
DLD-1	Gift from Dr Ahmet Hazini, University of Oxford
HEK293T	ATCC
KPC-F	Gift from Professor Jen Morton, Glasgow University
KPC-F;eGFP-OVA	Generated in house
KPC-F;mCherry	Generated in house
KPC-Y (2838c3)	Kerafast.
L929	Gift from Professor Ruth Muschel, University of Oxford
MC.38	Gift from Professor Ruth Muschel, University of Oxford
pMEL	ATCC
PSN-1	ATCC

The B3Z hybridoma cell line and DC2.4 were cultured in RPMI supplemented with 10% foetal bovine serum (FBS, Sigma-Aldrich), 100I.U./ mL penicillin (Gibco), 100µg/ mL streptomycin (Gibco), 1X non-essential amino acids (Gibco), 1mM sodium pyruvate (Gibco), 10mM HEPES pH 7.4 (Gibco), 50µM 2-mercaptoethanol (Gibco). All other cell lines outlined within this section were grown in high glucose DMEM supplemented with 100 I.U./ mL penicillin, 100µg/ mL streptomycin, and 10% FBS, henceforth referred to as complete DMEM. All cells in this study were cultured at 37ºC in 5% CO_2. All cell lines were negative for mycoplasma (Lonza Mycoalert™ test kit).

Transfection of cell lines

KPC-mCherry were generated from KPC-F using the jetPRIME transfection kit (Polyplus). Briefly, HEK293T cells were transfected with pLV-mCherry, pRRE, pRSV-Rev and pVSV-G in a 4:2:1:1 ratio, following manufacturer's protocol. After a 4hr incubation, the media was removed and replenished with complete DMEM. After 72hr, HEK293T were exhibiting red fluorescence and thus the

supernatant was harvested and passed through a 45µm filter. KPC-F seeded in a tissue culture-treated 6-well plate were cultured for 24 hours in 2mL of complete DMEM and 1mL of the HEK293T-transfected supernatant. The media was removed, cells were washed with room temperature PBS and the media replaced with a fresh 2mL of complete media and 1mL of supernatant. This was repeated until cells had received 3 viral hits. Cells were then expanded and sorted for mCherry+ cells by flow-assisted cell sorting (FACS). KPC-F;eGFP-OVA was generated using the same process outlined previously, transfecting pHR-Ova-IRES-eGFP and FACS performed on eGFP+ population. pHR-OVA-IRES-eGFP was a gift from Professor Simon Davies, all other plasmids were obtained from Addgene.

Animal models

Animal procedures were in accordance with UK Animal law (Scientific Procedures Act 1986), including local ethics approval. To establish orthotopic tumours, tumour cells (500 cells in 20µL 50:50 matrigel:PBS via 28G needle) were injected into the pancreas of 6-8 weeks female wild-type C57BL/6 mice via laparotomy. Tumour volume was monitored by ultrasound (Vevo 3100, FUJIFILM VisualSonics). Mice were randomly allocated to treatment groups when tumours reached 30mm^3. The PI3Kγ inhibitor IPI-549 (Selleckchem) was dissolved at 5% 1-methyl-2-pyrrolidinone (NMP) in polyethylene glycol 400 (PEG 400) and administered by oral gavage (15 mg/ kg) daily. Control groups received vehicle (5% NMP, 95% PEG) without the active drug. Anti-PD-1 antibody (250 µg per mouse, clone RPM1-14, Bio X Cell) was administered via intraperitoneal injection (IP) on days 0, 4, 7 and 10, anti-CSF-1 antibody (200 µg per mouse, clone 5A1, Bio X Cell) was administered via IP on day 0 and 3 times per week until end point, and anti-CD8a (250µg per mouse, Bio X Cell, clone 2.43) via IP on days 0, 3, 6 and 9. The MerTK inhibitor, UNC-2250 (Selleckchem), was reconstituted in DMSO to administer 25 mg/ kg (5% DMSO, 40% PEG 400, 5% Tween-20, 50% H_2O) via daily IP injections. Humane end-point was defined as tumour volume >500mm^3 or the development of symptoms (swollen abdomen, difficulties breathing, reduced mobility or other signs of ill health) wherein mice were culled by Schedule 1 method.

Genetically engineered mouse models

Pdx1-Cre; *LSL-Kras$^{G12D/+}$*; *LSL-Trp53$^{R172H/+}$* (KPC) mice were bred in house at the CRUK Scotland Institute, Glasgow, UK, in accordance with UK Home Office licence and approved by the University of Glasgow Animal Welfare and Ethical Review Board. The mice were maintained in a mixed background, housed in conventional environmentally enriched cages and given access to standard diet and water *ad libitum*. Genotyping of all mice was performed by Transnetyx (Cordoba, TN, USA). KPC mice were palpated weekly and the presence of a pancreatic tumour confirmed by 3D ultrasound imaging. Asymptomatic mice of both sex, in roughly equal proportions, were then randomly assigned to control or experimental group. Tumour volume was monitored by weekly 3D ultrasound imaging until the animal exhibited moderate clinical signs of pancreatic cancer (swollen abdomen, loss of body conditioning resembling cachexia, reduced mobility) when mice were culled by Schedule 1 method.

SARRP radiotherapy

Mice received stereotactic radiotherapy using the Small Animal Radiation Research Platform (SARRP200, XStrahl Life Sciences). Mice were injected IP with an iodine contrast agent (Omnipaque, GE healthcare, 20mg/ kg) and conebeam CT imaging was performed (720 slices) for each mouse and tumour localisation was confirmed. CT images were imported into Muriplan and used to select an isocenter. The tumuor was then irradiated using an arc (360 degrees) beam using an appropriately sized collimator at a dose rate of 3.9 Gy/minute.

Bioluminescence imaging of metastases

Mice bearing KPC-mCherry tumours receiving treatment as indicated were culled 30 days following tumour cell injection. The abdomen and chest cavities were opened immediately and the primary pancreatic tumour resected. Images were acquired using a Xenogen IVIS 200 imaging system (Caliper Life Sciences, Massachusetts, USA).

Lung isolation and metastases quantification

Immediately following culling, the trachea was dissected free, a longitudinal tracheotomy performed and a 23g needle inserted and secured using forceps. The lungs were insufflated with 5% low melting agarose (pre-warmed to 70ºC) and then immersed in Bouin’s solution. Macroscopic lung metastases were quantified manually. Lungs were subsequently processed, embedded, sectioned and stained with haematoxylin and eosin for histological confirmation.

Multiplex immunohistochemistry staining and image analysis

Multiplex immunofluorescence staining was carried out on 5 µm thick FFPE sections by the Translation Histopathology Laboratory (THL, University of Oxford). Briefly, sections were stained using the OPAL™ protocol (AKYOA Biosciences) on a Leica BOND RXm Auto-Stainer (Leica, Microsystems). Six consecutive staining cycles were performed using primary antibody-Opal fluorophore pairings (Ly6C/Ly6G (Gr-1) (Cell Signalling Technology Clone RB6-8C5); CD4 (1:500, ab183685; Abcam); CD8 (1:800, 98941; Cell Signaling); CD68 (1:1200, ab125212; Abcam); E-cadherin (1:500, 3195; Cell Signalling), CD161 (NK1.1). Primary antibodies were incubated for 30 minutes and detected using the BOND™ Polymer (Leica Biosystems) as per manufacturer's instructions. Sections were baked, dewaxed with BOND™ dewax solution, rehydrated with alcohol and incubated with Epitope Retrieval Solution 1 or 2 (ER1, ER2) (Leica Biosystems) at 100°C for 20-minutes Sections were washed X3 with BOND™ wash, blocked with peroxidase block (3 – 4% (v/v) hydrogen peroxide) for 5-minutes and subsequently washed 3X with BOND™ wash. Primary antibodies were incubated for 30-minutes, washed as before, and incubated with Anti-Rabbit Poly-HRP IgG for 8-minutes. Sections were washed twice in BOND™ wash, once in deionised water prior to Opal antibody incubation for 10-minutes. Sections were washed three times in deionised water and finally incubated with spectral DAPI (Akoya Biosciences) and slides mounted with VECTASHIELD® Vibrance™ Antifade Mounting Medium (Vector Laboratories). Whole slide multispectral images were obtained on the AKOYA Bioscience Vectra® Polaris™ (scanned at 20X magnification). Batch analysis and spectral unmixing of the tissues was performed with inForm 2.4.11 software. Batched analysed multispectral images were fused in HALO AI to produce a spectrally unmixed reconstructed whole tissue image.

Tissue dissociation and flow cytometry staining

Tumours were manually dissociated with a scalpel blade, incubated in Hank's balanced salt solution (Gibco) with 200I.U. Collagenase II (Worthington) and 1mg/ mL DNase I (Thermo Scientific) on a shaker at 37°C for 40 min and passed through a 70µm filter. For intracellular cytokine staining, protein transport inhibitor cocktail (eBioscience) was added to the dissociation solution. After blocking with anti-CD16/32, surface antigen staining was performed. For intracellular staining, the eBiosciences FOXP3 intra-cellular staining kit was used according to the manufacturer's instructions. Antibodies

used are listed in Appendix Table S1. Data were acquired on a Beckman Coulter Cytoflex. Data were analysed using FlowJo, version 10.9. Statistics performed using GraphPad Prism v10.4.0.

Generation of conditioned media

For the generation of L929 conditioned media (LCM), L929 cells were expanded in T175s until 70% confluent wherein the media was removed, cells were washed 3 times with PBS and provided 20mL of fresh complete DMEM. After 5 days, the media was removed, passed through a 0.22μm filter and stored at -20ºC until use.

For the generation of tumour conditioned media (TCM) or irradiated TCM (irTCM), KPC-F were expanded in T175 until 90% confluent wherein the media was removed, the cells washed 3 times with PBS and the cells replenished with 20mL of serum-free (SF) DMEM supplemented with penicillin and streptomycin. For irTCM, cells were then irradiated with 10Gy in a caesium-137 irradiator (GSR D1 from Gamma Service; dose rate 1.2 Gy/ min). After 24 hours, TCM or irTCM was harvested, passed through a 0.22μm filter and stored at -80ºC.

Additional tumour conditioned media, with and without irradiation, was obtained from other murine (MC38, B16F10 and KPC-Y) and human (DLD-1, A549, pMEL) cell lines as outlined above for efferocytosis assays only.

Bone marrow-derived macrophage culture, polarisation and tumour education

Bone marrow was harvested from the femurs of naïve C57BL/6 wild-type mice under sterile conditions. Cells were re-suspended in RPMI supplemented with 100I.U./ mL penicillin and 100mg/ mL streptomycin and 10% FBS, henceforth complete RPMI, with an additional 20% LCM and incubated for 5 days in non-tissue culture treated 100mm^2 plates (Greiner Bio-One). After 3 days the media was supplemented with an additional 3mL of 20% L929 conditioned media. At day 5-7 post bone marrow isolation, cells were harvested and counted after having been washed three times with room temperature PBS and lifted using TrypLE (Gibco).

M1 macrophages were obtained by culturing macrophages in SF RPMI supplemented with 20ng/ mL IFNγ and 100ng/ mL LPS (Peprotech) overnight, and M2 macrophages through culturing with SF

RPMI supplemented with 20ng/ mL each of IL-4 and IL-13 (Peprotech). Mock tumour educated macrophages (mTEMs) were obtained through culturing of macrophages with SF RPMI and 20% TCM, whilst irradiated tumour educated macrophages (irTEMs) were cultured with 20% irTCM. When assessing the impact of PI3Kγi on macrophage function cells were treated with 1µM of TG100-115 (Selleckchem), whilst controls cells were treated with 1µM of the vehicle DMSO (Thermo).

Myeloid-derived suppressor cell (MDSC) culture

Bone marrow was harvested from the femurs of tumour-bearing under sterile conditions. Cells were re-suspended in complete RPMI, supplemented with 40ng/ mL each of recombinant murine GM-CSF and recombinant murine IL-6 (PeproTech) and cultured in 100mm tissue cultured dishes. After 3 days, an additional 3mL of RPMI supplemented media was added. After 5 days the media was collected and the plates washed three times with PBS before being counted and used in further experiments. MDSCs were educated with TCM or irTCM as outlined above, with 1 µM DMSO vehicle or TG-110-115.

Human blood and pancreatic tumour processing

Human subjects were consented for blood and tissue collection following written informed consent. Tissue was collected under the University of Oxford Translational Gastroenterology Unit (TGU) biobank (v10). Research Ethics Committee number 21/YH/0206. For blood collection, 20mL of whole blood was collected in EDTA tubes (BD biosciences) and processed immediately. Recruited patients were planned for resectional surgery for pancreatic adenocarcinoma (pancreatoduodenectomy or distal pancreatectomy). At the point at which the specimen was extracted, a 5mm core punch biopsy was used to acquire a sample which was then immediately transferred to the laboratory for processing.

Tissue sections were prepared using the Leica Vibratome. Briefly, 5mm hole punch biopsies were taken and mounted in 5% low melting point agarose and sliced into 250µm sections. Slices were cultured in low glucose DMEM with 0.5% FBS, 1X Penicillin-Streptomycin-Neomycin antibiotic cocktail and amphotericin B (Gibco). Slices were either untreated, or subjected to either mock irradiation (0Gy) or irradiation (10Gy) alongside co-culture with 1µM TG100-115 or DMSO. Slices were cultured for 24 hours following treatment at 37ºC before being either stored in in RNALater (ThermoFisher) at -80ºC or fixed overnight in 10% neutral buffered formalin (Sigma-Aldrich) and left for 24 hours in 100%

ethanol before undergoing processing. Media was harvested, filtered and stored at -80ºC until needed.

Freshly collected blood from human subjects with pancreatic cancer was processed by density separation (Ficoll-Paquetm Plus Millipore, GE Healthcare). The peripheral blood mononuclear cell (PBMC) fraction was washed and CD14+ monocytes were isolated using a magnetic column (Miltenyi Biotec) and cultured in the presence of 20ng/ mL of recombinant human M-CSF (Peprotech). After 7 days, macrophages were harvested for use in assays.

Patient and Public Involvement

Patients or public were not involved in the design, conduct or reporting of our research. For future translational work we plan to include patients and/or public in the design of our research via a formalised patient and public engagement network.

Macrophage bioparticle phagocytosis assay

Bone marrow-derived macrophages were educated as described previously and seeded in a non-tissue culture treated 96-well plate 24 hours prior to the start of the experiment. For phagocytosis quantification the Vybrant™ phagocytosis assay kit (ThermoFisher) was used following the manufacturer's instructions.

Murine and human cell line efferocytosis assay

BMDMs were educated with mock or irradiated tumour conditioned media from KPC-F, KPC-Y, MC.38 or B16F10 ± PI3Kγi as previously outlined in a non-tissue culture treated 96-well plate. PBMC-derived macrophages from healthy donors were polarised likewise using tumour conditioned media from PSN-1, DLD-1, A549 and p53. To assess dendritic cell phagocytosis, the immortalised DC2.4 line was polarised using KPC-F generated TCM and irTCM only. Autologous cell lines were concurrently irradiated with 10 Gy. After 24 hours, cancer cells were labelled with carboxyfluorescein succinimidyl ester (CSFE, Thermo) as per manufacturer's protocol and incubated at a 4:1 ratio for 4 hours with their complementary polarised macrophages. After 4 hours, cancer cells were washed off, macrophages lifted, incubated with anti-CD16/32 and stained with conjugated antibodies against F4/80 for murine macrophages, CD11b for human macrophages, or CD11c and MHCII for dendritic cells and data obtained as outlined previously. Efferocytosis was quantified by measuring FITC+

population of antigen presenting cells by flow cytometry. Glycolysis was inhibited via co-administration of 2-deoxy-D-glucose (Cambridge Bioscience Ltd) during the 4-hour co-culture.

Patient derived efferocytosis assay

PBMC-derived macrophages from pancreatic patients were polarised for 24 hours using media collected from human precision cut tumour slices as outlined previously. PSN-1 cells were irradiated with 10Gy 24 hours prior, and then stained with CFSE according to the manufacturer's instructions. Tumour cells and macrophages were seeded at a 4:1 ratio and co-cultured for 4 hours. Tumour cells were removed by multiple PBS wash steps and macrophages dissociated enzymatically. Cells were incubated with anti-CD16/32 and stained with conjugated CD11b and data obtained as outlined previously. Efferocytosis was quantified by measuring FITC+ population of CD11b+ cells.

Antigen cross-presentation assay

Chicken ovalbumin peptide (Thermofisher Scientific) was covalently bonded to BioMag® beads (Bangs laboratories) as per the manufacturer's guidance and reconstituted in complete RPMI. Primary murine BMDMs and DC2.4 dendritic cells were exposed to TCM or irTCM ± 1μM TG100-115 for 24 hours. Ovalbumin bound beads were then added at a concentration of 20μg/ mL and incubated for 4 hours at 37°C. Beads were then washed off with PBS and B3Z T cells added at a ratio of 4:1 and incubated for 24 hours at 37°C. B3Z lysates were collected and β-galactosidase activity measured using the Promega β-Galactosidase Enzyme Assay System according to the manufacturer's instructions.

T cell suppression assay

CD8+ T cells were isolated from the spleens of naïve C57BL/6 wild-type mice using magnetic bead separation according to the manufacturer's instructions (Miltenyi Biotec). Prior to T cell suppression assay, 96-well plates were coated with 2μg/ mL anti-CD3/ anti-CD28. T cells were labelled with CSFE and resuspended in RPMI was supplemented with L-glutamine, 50μM β-mercaptoethanol and 500 I.U./ mL recombinant IL-2 (Peprotech), henceforth T cell media. Educated effector cells (BMDMs or

MDSCs) were added at the indicated ratios and co-cultured for 72 hours. CFSE signal in live T cells was analysed by flow cytometry and quantified using the FlowJo peak measurement software.

T cell killing, activation and suppression assays

Educated macrophages or MDSCs were co-cultured with irradiated KPC-F at a 1:4 ratio (APC:KPC-F) as outlined previously. After a 4-hour co-culture, KPC-F were washed off and naïve CD8+ T cells added at a 10:1 ratio (APC:CD8+). After 3 days, T cells were transferred to a fresh 96-well plate. KPC-F that had been irradiated 24 hours prior were added at a ratio of 10:1 (KPC-F:CD8+).

For the killing assay, after 72 hours, media was transferred to a fresh 96-well plate, the KPC washed three times with PBS before being lifted with TrypLE with the reaction stopped using the previously removed media. Cells were blocked with anti-CD16/32 and stained with CD11b, F4/80, CD8, Annexin V and PI. Data was acquired and analysed as previously discussed. KPC-F were defined as CD11b-F4/80-, CD8- and live, early apoptotic, late apoptotic and dead cells gated based on the Annexin V and PI fluorescence.

For the activation assay T cells were co-cultured with Golgi Stop and Golgi Plug (Biolegend) alongside KPC-F for 24 hours. The media, containing non-adherent CD8+ T cells, was transferred to a fresh 96-well plate where cells were blocked with anti-CD16/32 and stained with live/ dead marker, CD3, CD8, CD62L, CD44, CD69 before undergoing fixing and permeabalisation with Foxp3 / Transcription Factor Staining Buffer Set (Invitrogen). Cells were then stained with IFNγ. Data was acquired as previously described, with T cells gated using CD3 and CD8, with effector defined as CD44^{hi} CD62L^{lo}, memory cells as CD44^{hi} CD62L^{hi}, and naïve T cells as CD44^{lo}.

Protein isolation and Western blotting

Harvested cell pellets were lysed in Laemmli buffer (50 mM Tris-HCL [pH 6.8], 2% [w/v] sodium dodecyl sulphate, 1× protease Inhibitor cocktail, and 1× phosphatase inhibitor cocktail). Extracts were analysed by sodium dodecyl sulphate polyacrylamide gel electrophoresis using 4%–12% Bis-Tris NuPAGE gels (Invitrogen) and transferred onto polyvinylidene fluoride membranes (Millipore). After washing in PBS containing 1% Tween-20, membranes were blocked in 5% skim milk in PBS containing 1% Tween-20 and then incubated with the primary antibody overnight at 4°C (Total AKT, Cell Signalling Technology 2920, Phospho-AKT (Thr308) Cell Signalling Technology 9275). The

membranes were incubated with horseradish peroxidase–conjugated secondary antibodies (Cell Signalling Technologies) for 1 hour at room temperature and images acquired using the ChemiDoc imaging system (BioRad) after incubation with Thermo Scientific Pierce ECL or Amersham ECL (GE Healthcare). ImageJ software was used for the quantification of the bands. All bands were normalized against the loading controls.

Real-time quantitative PCR

RNA was extracted from samples using the Qiangen RNeAsy minikit according to the manufacturer's guidance. RNA samples were reverse-transcribed using Tetro cDNA Synthesis Kit (Thermo Fisher). A total of 25 ng of cDNA was loaded with SYBR Green (Thermo Fisher) and amplified in the following conditions: 40 cycles at 95°C for 15s, 60°C for 30s, and 72°C for 30s. mRNA expression and foldchange were analysed using the delta ct method, normalising for the housekeeping gene (β-actin).

Metabolite detection assays assays

Extracellular lactate was measured using the Lactate-Glo™ Assay (Promega). Metabolic assays were performed as per manufacturer's protocols. Unless stated otherwise, 20,000 cells were seeded per well and polarised using standard conditions previously outlined. After 24 hours, media was removed, and they were incubated for an additional 24 hours in low glucose SF DMEM. To obtain efferocytic populations, macrophages were co-cultured for 4 hours at a 4:1 ratio before KPC-F washed off and the macrophages incubated in low glucose SF DMEM for 24 hours before undergoing analysis.

Human scRNAseq analysis

Publicly available scRNA-seq data of pancreatic cancer tumours were collected from the following studies: Zhou et al [45]. "https://data.humantumoratlas.org", Werba et al [46]. "GSE205013", Peng et al [47]. (GSA: CRA001160), Steele et al [48]. " GSE155698", and Lin et al [49]. "GSE154778. The "Seurat" v4.3.0 R package was used for main scRNA-seq processing and analysis steps. Datasets across the five studies were independently processed for quality control before integration. Samples from each study were read using default parameters (min.cells = 10, min.features = 300) and merged. Cells with too low or high gene counts, high percentage mitochondrial gene expression (>15%) and contaminating

red blood cells (*HB* genes >10%) were filtered out from downstream analysis. Each dataset was normalized separately using the NormalizeData() Seurat function and the top 3000 highly variable genes used for integration were selected using FindVariableFeatures(), following by SelectIntegrationFeatures(). The datasets were then merged and converted to "anndata" file format required for scVI [50] integration from the "scvi-tools" Python package. An scVI model was created and trained with the default parameters and each patient sample was considered as an individual batch. SCVI latent embeddings were generated and used as input for UMAP visualization and neighbour identification. Clusters were found using the FindClusters() function with various resolutions (0.1-1) and cell types were labelled based on expression of canonical cell markers. Cell-Cell communication analysis was performed using the "CellChat" v2.0.0 R package [51]. Macrophage-related gene signatures from the literature were used to generate enrichment scores using the Seurat AddModuleScore() function.

RNA sequencing

For tumours, $1mm^3$ pieces were flash frozen in liquid nitrogen then homogenised in RLT buffer and RNA extracted using the RNEasy mini kit (Qiagen)according to the manufacturer's instructions. Myeloid cells (CD11b+) were isolated using magnetic beads separation (Miltenyi) according to the manufacturer's instructions. RNA was isolated immediately from cell pellets using the RNEasy micro kit (Qiagen). Preparation of RNA library and transcriptome sequencing was conducted by Novogene and results were provided as fastq files. These were uploaded into BaseSpace (Illumina) and processed using the DRAGEN RNA pipeline. The mouse reference genome for the alignment was built using the Dragen reference builder and the GRCm39 genome sequence. Differential gene expression analysis (DESeq2) was performed using the Dragen Differential Expression App, from which quant.sf files were downloaded and converted to .gct files. Gene expression, and clinical data were obtained via the "TCGAbiolinks" (2.24.3) R package for TCGA-PAAD dataset. Enrichment scores of the monocyte/macrophage gene signatures (top 10 differentially expressed markers) for each patient were calculated using the "GSVA" R package. Multivariate COX regression of the enrichment scores for disease-free survival was performed using the `ezcox` (1.0.2) package. Stage, age, sex, and radiation therapy were used as covariates. Heatmaps were generated using 'pheatmap' (1.0.12).

Packages/tools used for analysis RNAseq analysis:

Package (version)	Repository	Link to package/Publication
Seurat (4.3.0)	Github	https://satijalab.org/seurat/articles/install_v5.html
Scvi-tools	Python package index	https://pypi.org/project/scvi-tools/0.15.0/
TCGABiolinks (2.26.0)	Bioconductor	https://academic.oup.com/nar/article/44/8/
GSVA (1.46.0)	Bioconductor	https://bmcbioinformatics.biomedcentral.com/articles/10.1186/1471-2105-14-7
Ezcox (1.0.4)	Github	https://github.com/ShixiangWang/ezcox
CellChat (2.0.0)	Github	https://www.nature.com/articles/s41467-021-21246-9
